# Chemotaxis and Shorter O-Antigen Chain Length Contribute to the Strong Desiccation Tolerance of a Food-Isolated *Cronobacter sakazakii* Strain

**DOI:** 10.3389/fmicb.2021.779538

**Published:** 2022-01-04

**Authors:** Chengqian Qian, Min Huang, Yuhui Du, Jingjie Song, Huiqian Mu, Yi Wei, Si Zhang, Zhiqiu Yin, Chao Yuan, Bin Liu, Bin Liu

**Affiliations:** ^1^TEDA Institute of Biological Sciences and Biotechnology, Nankai University, Tianjin, China; ^2^Key Laboratory of Molecular Medicine and Biotherapy, School of Life Sciences, Beijing Institute of Technology, Beijing, China; ^3^Shenzhen Institute of Respiratory Diseases, The First Affiliated Hospital (Shenzhen People’s Hospital), Southern University of Science and Technology, Shenzhen, China; ^4^National Engineering Laboratory for Efficient Utilization of Soil and Fertilizer Resources, College of Resources and Environment, Shandong Agricultural University, Tai’an, China; ^5^Department of Sanitary Toxicology and Chemistry, School of Public Health, Tianjin Medical University, Tianjin, China; ^6^The Key Laboratory of Molecular Microbiology and Technology, Ministry of Education, Tianjin, China; ^7^Tianjin Key Laboratory of Microbial Functional Genomics, Tianjin, China

**Keywords:** *Cronobacter sakazakii*, desiccation, transcriptome, proteome, O-antigen chain length, chemotaxis

## Abstract

*Cronobacter sakazakii* is an opportunistic pathogen causing a lethality rate as high as 80% in infants. Desiccation tolerance ensures its survival in powdered infant formula (PIF) and contributes to the increased exposure to neonates, resulting in neonatal meningitis, septicemia, and necrotizing enterocolitis. This study showed that a food-isolated *C*. *sakazakii* G4023 strain exhibited a stronger desiccation tolerance than *C. sakazakii* ATCC 29544 strain. Considering the proven pathogenicity of G4023, it could be a big threat to infants. Transcriptome and proteome were performed to provide new insights into the desiccation adaptation mechanisms of G4023. Integrated analyses of these omics suggested that 331 genes were found regulated at both transcriptional and protein levels (≥2.0- and ≥1.5-fold, respectively). Deletion of chemotaxis system encoded genes *cheA* and *cheW* resulted in decreased tolerance in both short- and long-term desiccation. Reduced O-antigen chain length contributed to the biofilm formation and desiccation tolerance in the short term rather than the long term. In addition, biosynthesis of flagella, arginine and its transport system, and Fe/S cluster were also observed regulated in desiccated G4023. A better understanding of desiccation adaptation mechanisms of G4023 could in turn guide the operations during production and preservation of PIF or other food to reduce survival odds of G4023 and lower its exposure to get to infants.

## Introduction

*Cronobacter sakazakii* is a Gram-negative facultatively anaerobic pathogenic bacterium belonging to the family *Enterobacteriaceae*. Although the bacterium had been classified as *Enterobacter sakazakii* in 1980 (Farmer et al., 1980), it was later reclassified as a new genus, *Cronobacter*, containing seven species, namely, *C. sakazakii*, *Cronobacter malonaticus*, *Cronobacter muytjensii*, *Cronobacter turicensis*, *Cronobacter dublinensis*, *Cronobacter universalis*, and *Cronobacter condimenti* (Iversen et al., 2007, 2008; Joseph et al., 2012). Although the environmental reservoir of *C*. *sakazakii* is not evidently understood yet, reconstituted powdered infant formula (PIF) is reported to be the most associated vehicle for transmission (Chauhan et al., 2020; Parra-Flores et al., 2020). The desiccation tolerance of *C*. *sakazakii* ensures its survival in PIF and contributes to the increased exposure of neonates to the organism (Bennour Hennekinne et al., 2018). *C*. *sakazakii*-contaminated PIF has been epidemiologically linked to infections and illnesses and can cause neonatal meningitis, septicemia, and necrotizing enterocolitis in infants with a lethality rate as high as 80% (Yan et al., 2012; Almajed and Forsythe, 2016; Parra-Flores et al., 2018; Srikumar et al., 2019).

Bacteria can develop distinct strategies to tolerate desiccation stress. *Salmonella* can survive desiccation *via* the filamentation properties involving outer membrane antigen of lipopolysaccharides (LPSs), fimbriae, and cellulose (Gibson et al., 2006; White et al., 2006; Garmiri et al., 2008; Vanderlinde et al., 2010). *Listeria monocytogenes* was found to accumulate betaine, carnitine, and proline as osmolytes to cope with desiccation (Bayles and Wilkinson, 2000). The resistance of *C*. *sakazakii* to desiccation has been of research interest recently. A positive correlation between biofilm formation ability and desiccation tolerance was suggested, while the sequence type showed no significant correlation (Du et al., 2018). In addition, the physiological importance of trehalose in the desiccation survival of *C*. *sakazakii* cells has been confirmed (Srikumar et al., 2019). Although several factors have been reported influencing the desiccation tolerance of *C*. *sakazakii*, the pathways by which they regulate still lack sufficient experimental evidences.

Recently, the integration of transcriptome and proteome analyses has been an effective strategy to promote a better understanding of mechanisms in response to environmental stress (Lai et al., 2020). mRNA expression profile alone was insufficient for understanding the protein expression levels, since plenty of factors, such as RNA structure, regulatory proteins and sRNAs, codon bias, protein stability, protein degradation, and protein secretion could influence the level of protein expression (Maier et al., 2009; Haider and Pal, 2013; Wang X. et al., 2019; Buccitelli and Selbach, 2020). The analysis of mRNA and protein expression data usually failed to show a high correlation even under similar conditions (Chen et al., 2002; MacKay et al., 2004; Ghazalpour et al., 2011; Haider and Pal, 2013). Although RNA-seq and iTRAQ-based quantitative proteomics had been applied to reveal the potential desiccation resistance factors of *C*. *sakazakii* in recent years (Hu et al., 2017; Srikumar et al., 2019), the strains used and desiccation conditions were totally different between the two independent experiments. In order to gain a higher correlation between transcriptomic and proteomic data, and for a better understanding of desiccation tolerance mechanisms of *C*. *sakazakii*, we performed transcriptome and proteome analyses of *C*. *sakazakii* wild-type (WT) strain G4023 under the same desiccation conditions. G4023 is a food-isolated *C*. *sakazakii* strain that had been previously sequenced by us and experimentally proven to be associated with meningitis in neonates and infants (Wang M. et al., 2019). Considering label-free quantitation experiments show more accuracy and higher efficiency in quantitation of spiked-in standards than iTRAQ-labeled ones (Wang H. et al., 2012; Trinh et al., 2013; Sandberg et al., 2014), we chose the label-free quantitative proteome analysis in this study over the iTRAQ-labeled one.

## Results

### Genome, Biochemical Features, and Desiccation Tolerance Comparisons Between *Cronobacter sakazakii* G4023 and ATCC 29544

**FIGURE 1:**
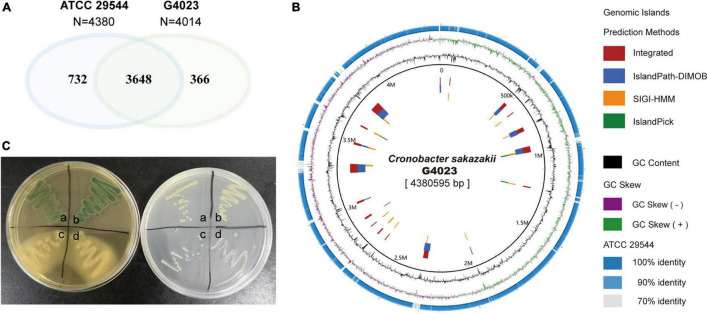
Genomic and biochemical comparisons between *C*. *sakazakii* G4023 and ATCC 29544. **(A)** Venn diagram shows the numbers of homologous and specific gene in genomes of *C*. *sakazakii* G4023 and ATCC 29544. **(B)** Genomic comparison of *C*. *sakazakii* G4023 and ATCC 29544 suggests the specific genes in G4023 mainly localized in genomic islands. **(C)** Bacteria colonies on DFI agar (left) and LB agar (right). ATCC 29544 forms green/blue colonies on DFI agar and produces yellow pigment on LB agar (a), which are identical to that of G4023 (b); *Salmonella* Typhimurium ATCC 14028 (c) and EHEC O157: H7 EDL933 (d) present white colonies on both agar mediums.

The WT *C*. *sakazakii* G4023, a food-isolated strain, has been proved to belong to sequence type 4 (ST4), and the pathogenicity has also been confirmed in our previous work (Wang M. et al., 2019). In this study, we compared the whole-genome sequence (WGS) of *C. sakazakii* G4023 with that of ATCC 29544. ATCC 29544 was clinically isolated and identified as a type strain of *C. sakazakii* (Farmer et al., 1980), and detailed information of both strains are summarized in Supplementary Table 1. The WGS comparisons showed there were 3,648 homologous genes, which account for 90.9 and 83.3% of total genes in G4023 and ATCC 29544, respectively (Figure 1A). Further analyses suggested the specific genes in G4023 were found mainly localized in genomic islands (Figure 1B), which were normally horizontally acquired to provided accessory genes for niche adaptation and pathogenicity (Schmidt and Hensel, 2004). In addition, whole-genome average nucleotide identity (ANI), average amino acid identity (AAI), and tetranucleotide usage deviation (TUD) between *C*. *sakazakii* G4023 and ATCC 29544 also demonstrated they belong to the same bacterial species (97.86%, 98.77%, and 0.999, respectively).

In addition to the genomic comparisons, biochemical identifications of *C*. *sakazakii* G4023 were also performed, with ATCC 29544 strain as a control. Both the typical features of *C*. *sakazakii*, green/blue colonies on selective Druggan Forsythe Iversen (DFI) agar and yellow pigment production on Luria–Bertani (LB) agar (Cawthorn et al., 2008), were observed in G4023 and ATCC 29544 bacteria; EHEC O157: H7 EDL933 and *Salmonella* Typhimurium ATCC 14028 bacteria presented white colonies on both DFI agar and LB agar (Figure 1C and Table 1). Meanwhile, a commercial kit for *C*. *sakazakii* identification was applied to G4023 bacteria, with ATCC 29544 as a control. Both of the strains reacted exactly like the typical *C*. *sakazakii* according to instruction (Table 1 and Supplementary Figure 1), which further proved that G4023 belongs to *C. sakazakii*.

**TABLE 1:** The results obtained from biochemical methods for the detection and identification of *Cronobacter sakazakii* G4023.

Strain	Phenotypic identification			1	2	3	4	5	6	7	8	9	10
	Yellow pigment	DFI agar	Oxidase test										
ATCC 29544	+	+	–	–	+	+	+	–	Gray	+	+	+	+
G4023	+	+	–	–	+	+	+	–	Gray	+	+	+	+

*1–10 represents mediums supplemented with different ingredients separately. 1, D-sorbitol; 2, L-rhamnose; 3, L-arginine dihydrolase; 4, ornithine decarboxylase broth; 5, lysine decarboxylase broth; 6, amino acid decarboxylase control; 7, D-sucrose; 8, D-melibiose; 9, amygdalin; 10, Simmons citrate.*

**FIGURE 2:**
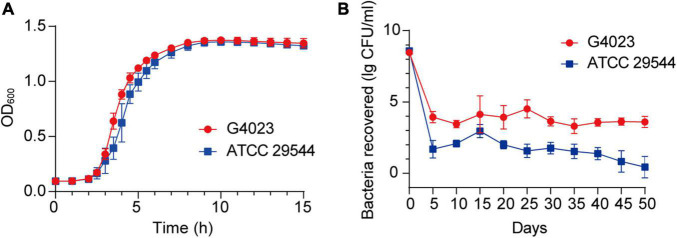
Growth curves and desiccation tolerance of *C*. *sakazakii* G4023 and ATCC 29544. **(A)** Growth curves of *C*. *sakazakii* G4023 and ATCC 29544 cultured in LB medium. The overnight cultured bacteria were diluted in the ratio of 1:1,000 (initial inoculum size was approximately 2 × 10^5^ CFU) and with shaking. OD600 measurements were performed every 10 min. Both strains entered the logarithmic phase at about 2 h and reached the stationary phase at about 9 h. **(B)** Progress curve of culturable cell counts during the desiccation of *C*. *sakazakii* G4023 and ATCC 29544. The CFU were counted every 5 days, and 50 days of desiccation tolerance was studied in total. ATCC 29544 barely survived, whereas quite a number of G4023 bacteria survived for at least 50 days in desiccation.

Growth curves of *C*. *sakazakii* G4023 and ATCC 29544 strains cultured in LB medium exhibited no significant differences [approximately 2 × 10^5^ colony forming units (CFU) were initially inoculated; Figure 2A]. To measure the desiccation tolerance of *C*. *sakazakii* G4023 strain, we desiccated the bacteria for up to 50 days and set *C*. *sakazakii* ATCC 29544 as a control. We dispensed the bacteria into individual wells of 12-well microtiter plates separately for desiccation treatment. Every 5 days, the viable count (CFU/ml) of each strain was documented to assess the desiccation tolerance ability. As shown in Figure 2B, viable counts of both G4023 and ATCC 29544 were sharply decreased during the first 5-day interval and tended to be stable after then. These results indicated that G4023 and ATCC 29544 strains might have adapted to desiccation during the first 5 days, and G4023 has better resistance to desiccation than ATCC 29544. Due to the strong desiccation tolerance and pathogenicity of G4023 (Wang M. et al., 2019), it could be a potential threat to infants. Therefore, we performed transcriptomic and proteomic analyses of desiccated *C*. *sakazakii* G4023 to provide insights into the desiccation adaption mechanisms.

### Overview of Transcriptomic Analyses

**FIGURE 3:**
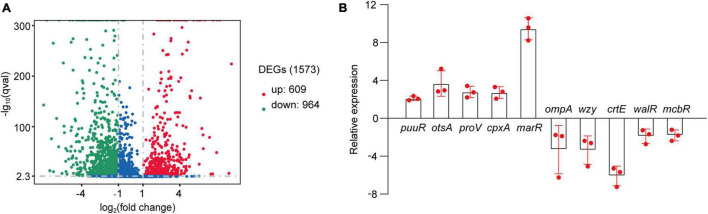
Transcriptome analysis. **(A)** Volcano plots of upregulated and downregulated (≥2-fold) mRNAs in desiccated *C*. *sakazakii* G4023. The red dots represent upregulated genes, green dots represent downregulated genes, and blue dots represent no significantly differentially expressed gene. **(B)** Confirmation of the RNA-seq results by qRT-PCR analysis; the expressions were normalized with the internal control gene *glnS*. The qRT-PCR fold-change tendencies agreed with the RNA-seq results. The error bars represent mean ± SD; *n* = 3.

As suggested above, *C*. *sakazakii* G4023 bacteria have adapted to desiccation during the first 5 days, and similar desiccation treatment for 4 days has been reported earlier; so samples used for RNA-seq and quantitative proteomic analyses were desiccated for 4 days as previously described (Shaker et al., 2008; Chaibenjawong and Foster, 2011). Bacteria cultured in LB medium were collected as the control group. High-throughput Illumina RNA-seq system (Novaseq 6000) was used to systematically catalogue the transcripts. After filtering, a total of 18,914,098 and 20,738,460 clean reads were obtained for the desiccated and control sets of *C*. *sakazakii* G4023, respectively. Approximately 98.11% of the total reads for desiccated *C*. *sakazakii* G4023 and 97.64% of those for control set were uniquely mapped to the respective reference genome. We observed 1,573 genes to be differentially expressed (≥2-fold change), out of which 609 were upregulated and 964 were downregulated (Figure 3A). Detailed differential expression profiles are shown in Supplementary Table 2.

The RNA-seq results were further confirmed by quantitative real-time PCR (qRT-PCR) analysis. The housekeeping gene *glnS* was used as the internal control gene as its expression did not change significantly under desiccation (Supplementary Figure 2). Ten genes were selected randomly to detect the fold-changes under desiccation. Although the fold-change values were not exactly alike, the regulation trends agreed with the RNA-seq results (Figure 3B), indicating the validity of the RNA-seq data.

**FIGURE 4:**
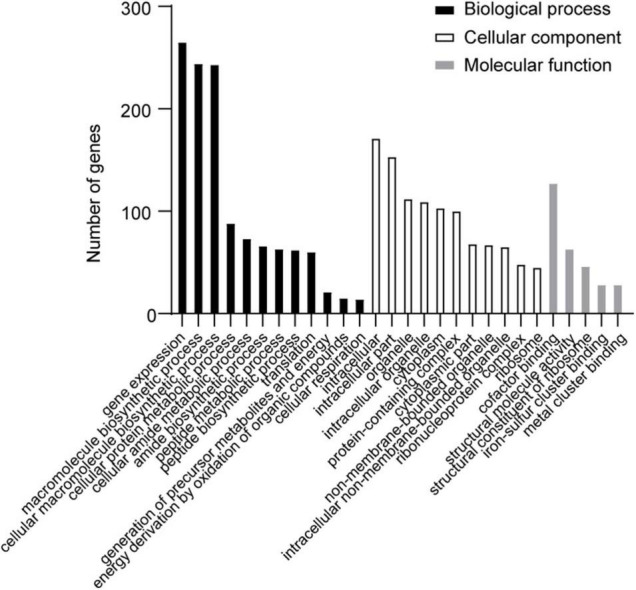
The most enriched 30 GO terms of desiccation *C*. *sakazakii* G4023. Twelve terms belong to biological process; eleven terms belong to cellular component, and five terms belong to molecular function.

The differentially expressed genes (DEGs) in the transcriptome were assigned annotations to three primary gene ontology (GO) domains: biological process (BP), cellular component (CC), and molecular function (MF). A total of 1,140 DEGs were successfully annotated against the GO database, of which 77 GO terms were significantly enriched (36 terms assigned to BP, 19 to CC, and 22 to MF, listed in Supplementary Table 2), and the most enriched 30 GO terms are shown in Figure 4. Genes involved with gene expression, macromolecule biosynthetic process, intracellular components, and cofactor binding were observed the most significantly differentially expressed in desiccated *C*. *sakazakii* G4023.

The DEGs were also subjected to Kyoto Encyclopedia of Genes and Genomes (KEGG) analysis. Only three pathways, namely, citrate cycle, oxidative phosphorylation, and carbon metabolism, were significantly enriched out of the 84 enriched ones (Supplementary Table 2), which suggested that the basal metabolisms were the most challenged in desiccated *C*. *sakazakii* G4023.

### Overview of Quantitative Proteomic Analyses

**FIGURE 5:**
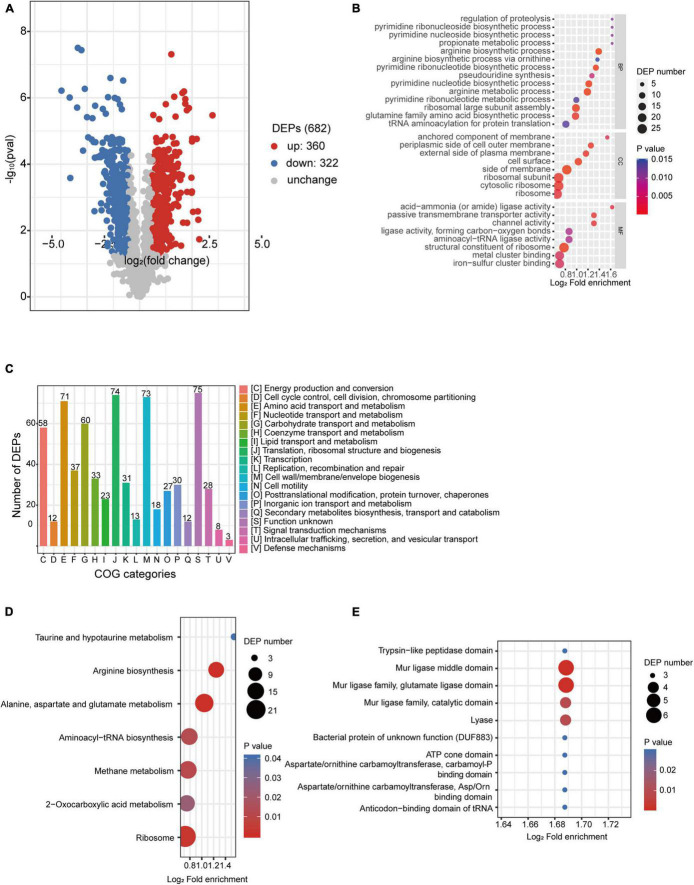
Proteomic analysis of desiccated *C*. *sakazakii* G4023. **(A)** Volcano plots of upregulated and downregulated (≥1.5-fold) proteins in desiccated *C*. *sakazakii* G4023. The red dots represent upregulated proteins, blue dots represent downregulated proteins, and gray dots represent no significantly differentially regulated protein. **(B)** COG analysis of DEPs. Bars represent the number of DEPs in each COG category. Besides the ones with unknown function, DEPs were categorized mostly in translation, ribosomal structure, and biogenesis, followed by in cell wall/membrane/envelope biogenesis and amino acid transport and metabolism. **(C)** Significantly enriched GO terms of DEPs in desiccated *C*. *sakazakii* G4023; 14 terms belong to BP, 8 terms belong to CC, and 8 terms belong to MF. **(D)** Seven KEGG pathways of DEPs were significantly enriched in desiccated *C*. *sakazakii* G4023. **(E)** Ten protein domains of DEPs were significantly enriched in desiccated *C*. *sakazakii* G4023.

Label-free quantitative proteomic analyses were simultaneously performed to investigate the regulations that happened at the protein level of desiccated *C*. *sakazakii* G4023, with LB-cultured bacteria set as the control group as well. Proteomics analysis showed that a total of 209,357 spectra were matched to 18,763 peptides, and 2,225 proteins were identified, out of which 1,754 were quantifiable. After data filtering (≥1.5-fold change), 360 proteins were observed upregulated and 322 were downregulated (Figure 5A). All the differentially expressed proteins (DEPs) are listed in Supplementary Table 3.

In order to obtain a better understanding of DEPs in desiccated *C*. *sakazakii* G4023, we performed the GO annotation and functional classification, subcellular location classification, and functional classification of clusters of orthologous groups of proteins (COG). Five hundred forty-seven identified DEPs (80.20% of the total) were annotated to GO terms (details shown in Supplementary Figure 3), and 30 hierarchically structured GO classes were significantly enriched (Figure 5B and Supplementary Table 3). According to the GO enrichment results, proteins related to proteolysis regulation, pyrimidine biosynthesis, propionate metabolic process, and acid-ammonia (or amide) ligase activity achieved the greatest fold enrichment. Subcellular location analysis of the 682 DEPs revealed that most of them (63%) are located in the cytoplasm, whereas outer membranal DEPs and extracellular DEPs were only 3.37 and 2.49%, respectively (Supplementary Figure 3). COG analyses showed that except for the functionally unknown proteins, DEPs were mostly categorized into translation, ribosomal structure and biogenesis (70/74 proteins were upregulated), cell wall/membrane/envelope biogenesis (50/73 proteins were downregulated), and amino acid transport and metabolism (49/71 proteins were upregulated) (Figure 5C and Supplementary Table 4). These indicated that a large abundance of proteins was needed by *C*. *sakazakii* G4023 to cope with desiccation, which was also revealed by Hu et al. (2017), and transport and metabolism of amino acids might contribute to the G4023 bacterial survival under desiccation stress.

Enrichment of KEGG pathways and protein domains of DEPs were performed as well. Three hundred sixty-three DEPs were allocated to the reference pathways in KEGG, and seven pathways were significantly enriched (*p* ≤ 0.05; Figure 5D and Supplementary Table 3). Three of the seven (42.86%) were related to biosynthesis and metabolism of amino acids (arginine, alanine, aspartate, glutamate, taurine, and hypotaurine). Correspondingly, amino acid biosynthesis and metabolism-related protein domains were also significantly enriched (Figure 5E and Supplementary Table 3).

### Overview of Integrated Analyses of Transcriptome and Proteome

**FIGURE 6:**
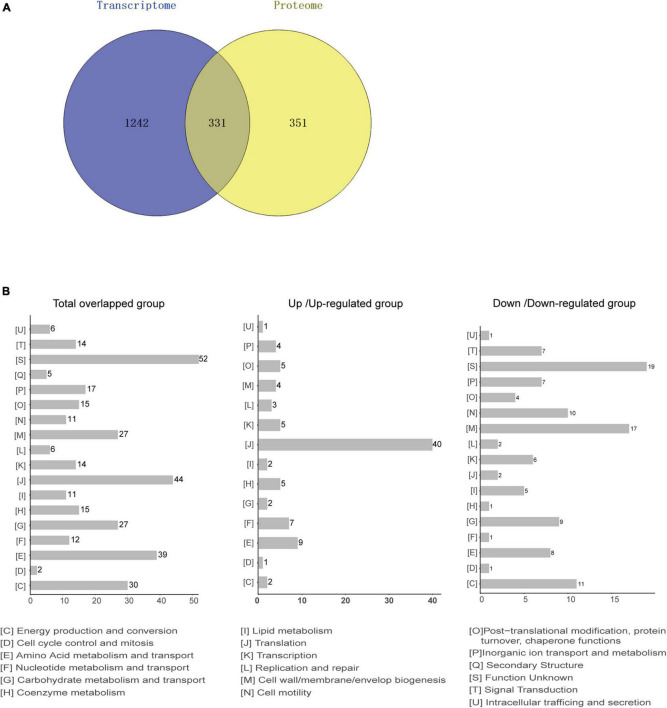
Integrated analysis of transcriptomic and proteomic data from desiccated *C*. *sakazakii* G4023. **(A)** Comparison between the numbers of differentially transcribed genes and differentially expressed proteins of desiccated *C*. *sakazakii* G4023. Genes with mRNA showing at least 2-fold change and proteins with at least 1.5-fold change are shown in the Venn diagram. In total, 1,573 DEGs and 682 DEPs were detected. The overlapped area represents 331 genes that were found differentially regulated at both transcriptional and protein levels. Adjusted *p* ≤ 0.05 for all data selected. **(B)** COG analyses of the 331 genes regulated at both transcriptional level and protein level (left); 44 were related to translation. In the up/upregulated group (genes were upregulated at both transcriptional and protein levels); genes were mostly categorized in translation (middle). In the down/downregulated group (genes were downregulated at both transcriptional and protein levels), genes were relatively related to the cell wall/membrane/envelop biogenesis (right).

Based on the transcriptome and proteome of desiccated *C*. *sakazakii* G4023, we performed an integrated analysis of these two omics to promote a better understanding of bacterial responses to desiccation. Overall, 331 genes were found to be regulated at both transcriptional level (≥2.0-fold) and protein levels (≥1.5-fold) under desiccation stress (Figure 6A). COG analysis of these 331 genes suggested that 325 genes were classified into 18 COG categories (Figure 6B, left). Besides those whose functions are unknown, genes were mostly involved in translation (44 genes), amino acid metabolism and transport (39 genes), energy production and conversion (30 genes), cell wall/membrane/envelope biogenesis (27 genes), and carbohydrate metabolism and transport (27 genes) categories, which were highly consistent with COG categorizing of DEPs. Further analysis suggested that 191 out of the 331 genes were regulated in matching tendencies, which were upregulated or downregulated at both transcriptional and protein levels (87 up/upregulated and 104 down/downregulated; Supplementary Table 5), and the fold changes are shown in the heatmap (Supplementary Figure 4). The COG classifications showed the up/upregulated group was dominantly sub-categorized into the translation category (Figure 6B, middle), and the down/downregulated group was mostly sub-categorized into the cell wall/membrane/envelope biogenesis.

### Chemotaxis System Favors Survival of *Cronobacter sakazakii* G4023 in Desiccation

In desiccated *C*. *sakazakii* G4023, protein levels of CheA, CheW, CheY, CheB, and CheR were upregulated 1. 78-, 1. 25-, 2. 00-, 3. 22-, and 1.23-fold, respectively, while the transcriptional expressions of the encoding genes (*cheA*, *cheW*, *cheB*, and *cheR*) were decreased (2. 22-, 3. 37-, 3. 54-, and 4.25-fold, respectively), and expression of gene *cheY* was not detected in transcriptome (Table 2). CheA is a histidine kinase and plays a central role in signal transduction of bacterial chemotaxis. The coupling protein CheW, cognate response regulator CheY, methyltransferase CheR, and methylesterase CheB are also involved (Kehry et al., 1984; Stephens et al., 2006; Muok et al., 2020).

**TABLE 2:** Transcriptional and protein expression changes of proteins involved in maltose/maltodextrin metabolism, ABC transporters, and arginine biosynthesis in desiccated *C*. *sakazakii* G4023.

Gene locus	Gene name	Protein accession	Protein description	Transcriptional expression fold change	Protein expression fold change
**Arginine biosynthesis relevant**					
LILKMKPI_03206	*argA*	A7MR21	Amino-acid acetyltransferase	3.58 (↓)	2.13 (↑)
LILKMKPI_00159	*argB*	A7ML88	Acetylglutamate kinase	3.39 (↓)	1.71 (↑)
LILKMKPI_00158	*argC*	A7ML82	N-acetyl-gamma-glutamyl-phosphate reductase	1.71 (↓)	3.62 (↑)
LILKMKPI_03766	*argD*	A7ME32	Acetylornithine/succinyldiaminopimelate aminotransferase	1.54 (↓)	2.29 (↑)
LILKMKPI_00157	*argE*	A7ML83	Acetylornithine deacetylase	2.02 (↓)	1.15(↓)
LILKMKPI_00481	*argF*	A7MIU6	Ornithine carbamoyltransferase	3.82 (↓)	4.70 (↑)
LILKMKPI_00160	*argG*	A7ML85	Argininosuccinate synthase	2.96 (↓)	2.23 (↑)
LILKMKPI_00161	*argH*	A7ML84	Argininosuccinate lyase	4.81 (↓)	2.08 (↑)
LILKMKPI_00477	*argI*	A7MQF6	Ornithine carbamoyltransferase	6.90 (↓)	3.31 (↑)
LILKMKPI_00475	*argR*	A7MQF9	Arginine repressor	1.34 (↓)	1.84 (↑)
LILKMKPI_00340	*argR*	A7MNR4	Arginine repressor	1.54 (↓)	1.14 (↑)
LILKMKPI_00639	*carA*	A7MI93	Carbamoyl-phosphate synthase small chain	2.60 (↑)	1.88 (↑)
LILKMKPI_00640	*carB*	A7MIA6	Carbamoyl-phosphate synthase large chain	1.32 (↑)	2.63 (↑)
**Flagellar and chemotaxis relevant**					
LILKMKPI_01587	*flgM*	A7MG38	FlgM domain-containing protein	1.12 (↓)	2.59 (↑)
LILKMKPI_02465	*fliA*	A7MJH6	RNA polymerase sigma factor	4.43 (↓)	1.86 (↑)
LILKMKPI_01598	*flgK*	A7MFQ0	Flagellar hook-associated protein 1	5.76 (↓)	7.87 (↓)
LILKMKPI_01599	*flgL*	A7MFP9	Flagellin and related hook-associated proteins	2.89 (↓)	2.68 (↓)
LILKMKPI_02478	*fliC*	A7MJI4	Flagellin and related hook-associated proteins	7.62 (↓)	2.02 (↓)
LILKMKPI_02479	*fliD*	A7MJI3	Flagellar hook-associated protein 2	4.35 (↓)	10.10 (↓)
LILKMKPI_02499	*fliG*	A7MJJ3	Flagellar motor switch protein	1.49 (↑)	1.44 (↑)
LILKMKPI_02505	*fliM*	A7MJK4	Flagellar motor switch protein FliM	2.54 (↓)	2.03 (↑)
LILKMKPI_02506	*fliN*	A7MJK3	Flagellar motor switch protein FliN	1.59 (↑)	2.86 (↑)
LILKMKPI_02428	*cheA*	A7MED8	Chemotaxis protein CheA	2.22 (↓)	1.78 (↑)
LILKMKPI_02427	*cheW*	A7MED9	CheW-like domain-containing protein	3.54 (↓)	1.25 (↑)
LILKMKPI_02417	*cheY*	A7MED1	Response regulatory domain-containing protein	NA	2.00 (↑)
LILKMKPI_02418	*cheB*	A7MED0	Protein-glutamate methylesterase/protein-glutamine glutaminase	3.37 (↓)	3.22 (↑)
LILKMKPI_02419	*cheR*	A7MEC9	Chemotaxis protein methyltransferase	4.25 (↓)	1.23 (↑)
**Iron-sulfur cluster relevant**					
cLILKMKPI_02963	*iscS*	A7MGX6	Cysteine desulfurase IscS	9.2 (↑)	2.31 (↑)
LILKMKPI_02962	*iscU*	A7MGX9	Iron-sulfur cluster assembly scaffold protein IscU	6.09 (↑)	2.06 (↑)
LILKMKPI_02961	*iscA*	A7MGY0	Iron-binding protein IscA	6.40 (↑)	NA
LILKMKPI_02960	*hscB*	A7MGY1	Co-chaperone HscB	7.27 (↑)	NA
LILKMKPI_02959	*hscA*	A7MGW6	Chaperone protein HscA	5.21 (↑)	1.96 (↑)
LILKMKPI_01746	*sufA*	A7MF63	Fe-S_biosyn domain-containing protein	3.59 (↑)	2.00 (↓)
LILKMKPI_01747	*sufB*	A7MF62	Cysteine desulfurase activator complex subunit SufB	4.43 (↑)	1.32 (↓)
LILKMKPI_01748	*sufC*	A7MF61	ABC transporter domain-containing protein	2.71 (↑)	1.89 (↓)
LILKMKPI_01749	*sufD*	A7MF60	Cysteine desulfurase activator complex subunit SufD	3.72 (↑)	1.22 (↓)
LILKMKPI_01750	*sufS*	A7MF59	Cysteine desulfurase	2.83 (↑)	1.47 (↓)
LILKMKPI_01751	*sufE*	A7MF58	Cysteine desulfuration protein SufE	0.82 (↓)	1.18 (↑)
**Lipopolysaccharide synthesis relevant**					
LILKMKPI_00368	*lptA*	A7MJE3	Lipopolysaccharide export system protein	1.93 (↓)	2.46 (↑)
LILKMKPI_02206	*lapB*	A7MME4	Lipopolysaccharide assembly protein B	1.06 (↓)	1.3 (↓)
LILKMKPI_02207	*lapA*	A7MMG0	Lipopolysaccharide assembly protein A	1.14 (↓)	NA
LILKMKPI_03986	*waaC*	A7MQ89	Lipopolysaccharide heptosyltransferase I	1.89 (↓)	1.65 (↓)
LILKMKPI_02563	*wbbL*	NA	Glycosyl transferase family 2	6.76 (↓)	NA
LILKMKPI_02564	*orf1*	NA	Glycosyl transferase	13.46 (↓)	NA
LILKMKPI_02565	*orf2*	NA	Glycosyl transferase	7.73 (↓)	NA
LILKMKPI_02566	*wzy*	NA	Oligosaccharide repeat unit polymerase	12.08 (↓)	NA
LILKMKPI_02567	*orf3*	NA	Glycosyl transferase	5.65 (↓)	NA
LILKMKPI_02568	*orf4*	NA	Glycosyl transferase	7.16 (↓)	NA
LILKMKPI_02569	*wzx*	NA	O-antigen flippase	4.98 (↓)	NA
LILKMKPI_02570	*rmlC*	NA	dTDP-4-keto-6-deoxy-Dglucose3, 5-epimerase	7.75 (↓)	NA
LILKMKPI_02571	*rmlA*	A7MHE4	Glucose-1-phosphate thymidylyltransferase	3.90 (↓)	NA
LILKMKPI_02572	*rmlD*	NA	dTDP-6-deoxy-L-mannose-dehydrogenase	2.85 (↓)	NA
LILKMKPI_02573	*rmlB*	A7MHE3	dTDP-glucose 4,6-dehydratase	4.46 (↓)	1.38 (↑)
LILKMKPI_02561	*wzzB*	A7MHD9	Wzz domain-containing protein	1.70 (↓)	1.72 (↓)
LILKMKPI_03236	*fepE*	A7MR50	Wzz domain-containing protein	2.67 (↓)	1.74 (↑)

**FIGURE 7:**
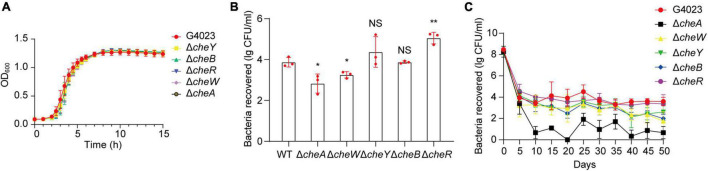
Desiccation tolerance of chemotaxis gene mutant strains of *C*. *sakazakii* G4023. **(A)** Growth curves of *C*. *sakazakii* WT strain G4023 and chemotaxis gene-mutant strains (G4023Δ*cheY*, G4023Δ*cheB*, G4023Δ*cheR*, G4023Δ*cheW*, and G4023Δ*cheA*) cultured in LB medium with shaking; OD600 measurements were done every 10 min. There was no growth deficiency in the *che* mutant strains compared to that in WT. **(B)** Re-cultured cell counts (CFU/ml) of *C*. *sakazakii* WT strain G4023 and the chemotaxis gene-mutant strains (G4023Δ*cheY*, G4023Δ*cheB*, G4023Δ*cheR*, G4023Δ*cheW*, and G4023Δ*cheA*) in LB agar medium after being desiccated for 4 days. The error bars represent mean ± SD; *n* = 3. Differences between two groups were evaluated by a two-tailed Student’s *t*-test. A *p*-value of < 0.05 was considered statistically significant. Asterisks are used to indicate significant differences (*p < 0.05; **p < 0.01; NS, not significant). **(C)** Progress curve of culturable cell counts during the desiccation of WT G4023, G4023Δ*cheY*, G4023Δ*cheB*, G4023Δ*cheR*, G4023Δ*cheW*, and G4023Δ*cheA*. The CFU were counted every 5 days, and 50 days of desiccation tolerance was studied in total.

To further investigate what role *che* system plays in desiccated *C*. *sakazakii* G4023, we constructed *cheA*, *cheW*, *cheY*, *cheB*, and *cheR* mutant strains of G4023, and their growth rates exhibited no significant differences *in vitro* (Figure 7A). Desiccation tolerance tests were then performed. Four-day desiccation results suggested that the G4023Δ*cheR* strain exhibited stronger desiccation tolerance than *C*. *sakazakii* G4023, while G4023Δ*cheA* and G4023Δ*cheW* showed significantly decreased tolerance (Figure 7B). The 50-day desiccation tests showed that G4023Δ*cheA*, G4023Δ*cheW*, and G4023Δ*cheB* exhibited significantly decreased resistance to desiccation compared with that of *C*. *sakazakii* G4023, especially G4023Δ*cheA* (Figure 7C). These results together with the upregulations in CheAWYBR protein levels suggested that the chemotaxis signaling cascade, especially the core protein CheA, might be important in the desiccation survival of *C*. *sakazakii* G4023.

### Flagellar Biosynthesis Is Stimulated in Desiccated *Cronobacter sakazakii* G4023

It has been reported that flagellum synthesis can be regulated by a chemotaxis signal transduction pathway to optimize swim cell–swarm cell differentiation in response to changing environmental conditions in *Rhodospirillum centenum* (Berleman and Bauer, 2005). Since the chemotaxis system relevant proteins were upregulated in desiccated *C*. *sakazakii* G4023, we wondered whether the flagellar synthesis was affected as well. Flagellar biosynthesis genes are organized in a three-tiered cascade allowing for regulated expression (Romilly et al., 2020). The products of class I operon, FlhD and FlhC, are regulators positively controlling the expression of class II operons. However, the transcriptional expressions of genes *flhD* and *flhC* showed no significant changes (0.89- and 1.01-fold), and regulations at protein levels were not determined as only FlhD was detectable in the desiccated sample, which could result from the fact that the protein amount is not enough to reach the detection threshold of label-free quantitative proteome analysis.

Although expression regulations of class I operon were not suggested in the omics analyses, FliA and FlgM encoded by class II genes were found upregulated 1.86- and 2.59-fold, respectively at protein level; transcriptional expressions of *fliA* and *flgM* genes were downregulated 4.43- and 1.12-fold, respectively. FliA is a sigma factor σ^28^, whereas FlgM is an anti-sigma factor which binds to FliA to prevent its association with RNA polymerase core enzyme (Hughes and Mathee, 1998). The FliA–FlgM regulatory system is involved not only in the expression of class III but also in that of class II operons, and the relative concentration of FliA and FlgM may play an important role in the production of flagella (Kutsukake and Iino, 1994). In addition to these two regulators, class II genes also encode basal body and hook components (Kapatral et al., 1996). Unlike the upregulations of FliA and FlgM, the hook-associated proteins FlgK, FlgL, FliC, and FliD were all found downregulated over 2-fold at both transcriptional and protein levels (Table 2). The downregulation of basal body and hook components of flagella suggested that flagellum demand in *C*. *sakazakii* G4023 was decreased in desiccation, and energies could be saved for better survival.

Class III genes regulated by FliA–FlgM regulatory system encode flagellar motor switch proteins and flagellin, such as FliG, FliM, and FliN, which were found increased 1. 44-, 2. 03-, and 2.86-fold, respectively, at protein level (Table 2). It has been reported that FliG, FliM, and FliN interact with one another to form a switch complex, which in turn is thought to interact with chemotaxis protein CheY, to induce clockwise rotation of the flagellar motors and tumbling of the cell (Toker and Macnab, 1997). From these results, we guessed that when *C*. *sakazakii* G4023 bacteria encounter the desiccation, flagellum numbers needed by each cell is decreased, but the flagellar motors and tumbling ability of bacteria are needed to be increased to favor survival in desiccation stress.

### Reduced O-Antigen Chain Length Contributes to the Biofilm Formation and Aids the Bacterial Survival of Desiccated *Cronobacter sakazakii* G4023 in Short Term

LPS, which is composed of lipid A, a core oligosaccharide, and O-antigen repeats (Liu et al., 2019), anchors in the outer membrane of gram-negative bacteria and serves as an effective barrier between the bacterium and its environment (Sun et al., 2011). It has been reported that cleavage of LPS offered *C*. *sakazakii* bacteria higher resistance to the hostile environment as it caused outer membrane defects and increased biofilm formation (Wang L. et al., 2012).

In this study, we focused on the O-antigen, which is generally not necessary for the survival of the microorganism (Gronow and Brade, 2001), to investigate its role in adaptation to desiccation stress of *C*. *sakazakii* G4023. The O-antigen biosynthesis genes in the genome of *C*. *sakazakii* are normally located between *galF* and *gnd* (Sun et al., 2011), and the transcriptional expressions of these genes were found downregulated in desiccated *C*. *sakazakii* G4023 (Table 2). However, most of the proteins encoded by these genes were not able to be quantified at the protein level; this may be due to their low abundance as they were mostly located on cell membranes (Okuda et al., 2016). We tried to mutate the O-antigen biosynthesis essential gene, *wzy* or *wzx* (Wang et al., 2009), in *C*. *sakazakii* G4023 but failed; we then turned to two other important genes for O-antigen biosynthesis, *wzzB* and *fepE*, which are responsible for the biosynthesis of long-chain and very-long-chain O-antigens, respectively (Murray et al., 2003). O-antigen chain length has been reported to affect survival and infection procedures in several bacteria (Murray et al., 2003; Kintz and Goldberg, 2011; da Silva et al., 2018). The expression of *wzzB* in this study was found decreased 1.70- and 1.72-fold at transcriptional and protein levels, respectively; while *fepE* was found downregulated 2.67-fold at transcriptional level but upregulated 1.74-fold at protein level.

**FIGURE 8:**
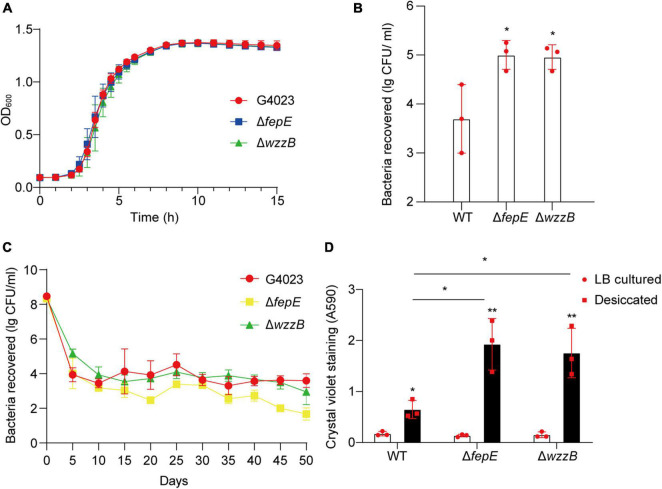
The role of O-antigen chain length-regulation genes in the desiccation tolerance of *C*. *sakazakii* G4023. **(A)** Growth curves of *C*. *sakazakii* wild-type (WT) strain G4023, G4023Δ*fepE*, and G4023Δ*wzzB* in LB medium at 37°C with shaking; OD600 measurements were done every 10 min. There were no differences in the growth of G4023, G4023Δ*fepE*, and G4023Δ*wzzB*. **(B)** Re-cultured cell counts (CFU/ml) of *C*. *sakazakii* WT strains G4023, G4023Δ*fepE*, and G4023Δ*wzzB* in LB agar medium after being desiccated for 4 days. All of the three strains could survive desiccation, and the desiccation tolerance of G4023Δ*fepE* and G4023Δ*wzzB* was significantly stronger than that of wild-type strain. **(C)** Progress curve of culturable cell counts during the desiccation of WT G4023, G4023Δ*fepE*, and G4023Δ*wzzB*. The CFU were counted every 5 days, and 50 days of desiccation tolerance was studied in total. **(D)** Absorbances of crystal violet at 590 nm was used to assess the biofilm formation abilities of *C*. *sakazakii* WT strain G4023, G4023Δ*fepE*, and G4023Δ*wzzB*. Biofilm formation of LB cultured strains and desiccation-treated strains were detected separately. All the three strains showed significantly increased biofilm formation under desiccation stress. Desiccated G4023Δ*fepE* and G4023Δ*wzzB* formed more biofilms than the desiccated WT strain, although the three showed no significant difference in biofilm formation when cultured in LB medium. The error bars represent mean ± SD; *n* = 3. Differences between two groups were evaluated by a two-tailed Student’s *t*-test. A *p*-value of < 0.05 was considered statistically significant. Asterisks are used to indicate significant differences (*p < 0.05; **p < 0.01).

To further investigate whether FepE and WzzB played roles in desiccation tolerance of *C*. *sakazakii* G4023, we constructed the *fepE* and *wzzB* mutant strains (G4023Δ*fepE* and G4023Δ*wzzB*). Both mutant strains showed no growth defects *in vitro* (Figure 8A), and the desiccation tolerance tests were performed. Results of a 4-day term of desiccation showed that both G4023Δ*fepE* and G4023Δ*wzzB* exhibited better survival than G4023 (Figure 8B), whereas the survival advantages faded with desiccation time, especially G4023Δ*fepE*. By the 50th day of desiccation, bacterial recovery counts of G4023Δ*fepE* were significantly less than those of G4023 (Figure 8C). These results indicated that shorter O-antigen chain length biosynthesis aids in the survival of *C*. *sakazakii* G4023 in the short-term rather than the long-term desiccation.

Moreover, crystal violet staining assays were also performed to quantify the biofilm formed by G4023, G4023Δ*fepE*, and G4023Δ*wzzB*. There was no significant difference in biofilm formation between G4023 and the mutant strains when they were cultured in LB. However, after desiccation treatment for 4 days, all of them exhibited significant increases in biofilm formation. Notably, biofilm formed by desiccated G4023Δ*fepE* and G4023Δ*wzzB* was significantly more than that in desiccated G4023 (Figure 8D). These results indicated that *C*. *sakazakii* G4023 increased its biofilm formation to overcome desiccation, and shortening of O-antigen chain length could further facilitate the biofilm formation yet only happened when bacteria were suffering desiccation.

### Biosynthesis and Transport of Arginine Are Upregulated in Desiccated *Cronobacter sakazakii* G4023

COG and KEGG enrichment analyses of DEPs, as well as COG classifications of genes regulated at both transcriptional and protein levels, suggested that proteins related to transport and metabolism of amino acids were significantly regulated in desiccated *C*. *sakazakii* G4023. Detailed analyses revealed that proteins involved in arginine biosynthesis, such as ArgABCDEFGH, CarAB, and the regulator ArgR (Caldara et al., 2008), were upregulated; furthermore, ArtI and ArtJ involved in arginine ABC transporter (Bai et al., 2021) were also upregulated 1.7- and 2.22-fold at protein level, respectively (Table 2). These results indicated that biosynthesis and transport of arginine may contribute to the survival of *C*. *sakazakii* G4023 in desiccation, but whether and how the arginine functions required further investigations.

During the analyses, we found the transcriptional expressions of arginine biosynthesis genes (*argABCDEFGH*) were downregulated (Table 2). As it has been reported that productions of all enzymes involved in arginine biosynthesis are subjected to repression by arginine, mediated by the repressor ArgR (Vyas and Maas, 1963), these downregulations could have resulted from the upregulated protein level of ArgR.

### Iron/Sulfur Biosynthesis Is Influenced in Desiccated *Cronobacter sakazakii* G4023

Iron/sulfur cluster is involved in all kingdoms of life to be catalysts or redox sensors in diverse BPs and is formed by Fe/S biogenesis systems NIF, ISC, and SUF (Yang et al., 2006; Roche et al., 2013), of which ISC and SUF systems allow the maturation of all Fe/S proteins in cells, while NIF is dedicated to the maturation of nitrogenase (Ayala-Castro et al., 2008). Genes encoding ISC and SUF are present in the genome of *C*. *sakazakii* G4024, while those for NIF are absent. The operon *iscSUA-hscBA*, encoding the ISC system (Roche et al., 2013), were observed upregulated 9. 2-, 6. 09-, 6. 40-, 7. 27-, and 5.21-fold, respectively, at the transcriptional level in desiccated *C*. *sakazakii* G4024. Meanwhile, protein amounts of IscS, IscU, and HscA were also increased 2. 31-, 2. 06-, and 1.96-fold, respectively; whereas IscA and HscB were not detectable in proteome (Table 2). Additionally, genes encoding the two sub-complexes, SufBCD and SufSE, of SUF system were found transcriptionally regulated (*sufABCDS* were increased 3. 59-, 4. 43-, 2. 71-, 3. 72-, and 2.83-fold, respectively, while *sufE* was decreased 1.22-fold). Besides, the protein levels of SufABCDSE were 0. 50-, 0. 76-, 0. 53-, 0. 82-, 0. 68-, and 1.18-fold, respectively, in desiccated *C*. *sakazakii* G4023 compared with that in LB-cultured (Table 2). Considering the ISC systems were upregulated at both transcriptional and protein levels and the SUF system was increased at the transcriptional level but decreased at the protein level, we speculated that when *C*. *sakazakii* G4023 suffered desiccation, the bacteria would prefer the ISC system instead of the SUF to synthesize F/S cluster to cope with the stress. However, these surmises need to be confirmed with further experimental investigations.

## Discussion

The goals of this study were to investigate the desiccation tolerance of *C*. *sakazakii* G4023 and reveal potential factors involved through the analyses of transcriptome and proteome. This study suggested *C*. *sakazakii* G4023 strain has stronger desiccation tolerance than *C*. *sakazakii* ATCC 29544 strain. This makes sense as G4023 is a food-isolated strain, while ATCC 25544 was a clinical one. Food-isolated microorganisms are exposed to a wide range of stresses, such as desiccation and osmotic stress, in food processing or preparation environments (Osaili and Forsythe, 2009), so G4023 must be equipped with strong desiccation tolerance to survive. Additionally, ATCC 29544 was reported to have a weak ability to form biofilm (Yang et al., 2016), while biofilm formation has been proved to contribute to desiccation resistance in *C. sakazakii* as well as other species of microorganism (Hansen and Vogel, 2011; Du et al., 2018). Therefore, the weaker desiccation tolerance of ATCC 29544 is reasonable.

iTRAQ-based comparative proteome and RNA-seq-based transcriptome were performed in 2017 and 2019, respectively, to gain insights into responses in C. sakazakii to desiccation (Hu et al., 2017; Srikumar et al., 2019). However, the *C*. *sakazakii* ATCC 29544 strain was used in the proteomic analyses and only desiccated for 24 h. Since the stronger desiccation tolerance of G4023 than ATCC 29544 was suggested in this study, using G4023 instead of ATCC 29544 to reveal desiccation resistance mechanisms seems more convincing. Additionally, although a PIF-isolated *C*. *sakazakii* SP291 strain was used in the transcriptional analyses study, the desiccated time was only 4 h, and it seems too short to reveal the regulations that happened during the long-term desiccation. So in this study, *C*. *sakazakii* G4023, which has stronger resistance to desiccation, was treated under dry stress for 4 days to perform the RNA-seq and protein quantification.

Subcellular location analyses of DEPs in this study showed that only 3.37 and 2.49% were outer membranal and extracellular proteins, respectively. The low percentages may be due to the low abundance of these proteins in samples, as solubilization, sample handing, preparation, separation, and analysis of the membranal proteins are usually complicated due to their hydrophobic nature (Han et al., 2008). Therefore, most of the proteins involved in O-antigen biosynthesis in G4023 were not able to be quantified as they were mostly located on cell membranes. Several methodologies have been developed to improve membrane protein identification and also to perform reliable quantification, which allows in-depth membrane protein studies (Helbig et al., 2010). Consequently, the membrane proteomic analysis of desiccated *C*. *sakazakii* G4023 could be performed in the future to gain further insights into the roles of O-antigen and other membranal proteins playing under desiccation stress.

Integrated analyses of transcriptome and proteome revealed that a large number of genes related to translation were upregulated at both transcriptional and protein levels in this study. Yang et al. (2016) suggested previously that *C*. *sakazakii* with a stronger biofilm formation ability contains higher levels of proteins related to 50S ribosome, 30S ribosome, and purine and pyrimidine biosynthesis. Therefore, the upregulated translation-relevant proteins may assist the biofilm formation in desiccated *C*. *sakazakii* G4023, which in turn contributes to adaptation and survival. Although this study has experimentally proved increased biofilm formation in G4023 under desiccation, the detailed mechanisms still need to be revealed in the future. Meanwhile, the observed downregulation of cell wall/membrane/envelope biogenesis genes at both transcriptional and protein levels in this study also makes sense because bacterial surface structures bear the brunt of desiccation. Instead of re-building the original surface structures, the re-organizations, such as biofilm formation, could endow *C*. *sakazakii* bacteria with better survival during desiccation (Li et al., 2012). So biogenesis of cell wall/membrane/envelope may be reduced in desiccated G4023. At the same time, the desiccated situation is harsh for bacteria to survive, and the limited energy will prioritize BPs that are more conducive to survival.

As opposite regulation trends of the chemotaxis genes were observed in transcriptome and proteome of desiccated *C*. *sakazakii* G4023, there may exist negative feedback regulations on the expression of *che* operons, which require further investigations. Furthermore, since 416 chemosensory systems within 245 prokaryotic species have been revealed (Wuichet and Zhulin, 2010), and several gram-negative species have been reported to utilize variations of chemotaxis signaling cascade to switch lifestyles, such as flagellar motility, type IV pili-based motility, cell development, biofilm formation, exopolysaccharide production, and flagellum biosynthesis, to survive environmental stress (He and Bauer, 2014), it is likely that *C*. *sakazakii* G4023 could also utilize a chemosensory system to switch lifestyles for better survival in desiccation.

The decreased desiccation resistance of G4023Δ*fepE* in the long term and the upregulated protein level of FepE suggest that FepE may play a role in the survival of *C*. *sakazakii* G4023 under desiccation stress. In view of the fact that FepE was also involved in ferric enterobactin transportation in Enterobacteriaceae bacteria (Ozenberger et al., 1987), we speculated that deficiency in *fepE* might result in impaired ferric enterobactin transport, which could be disadvantageous in the long-term desiccation tolerance of *C*. *sakazakii* G4023.

We are just starting to understanding the influences of the chemotaxis system and the O-antigen chain length on desiccation tolerance of *C*. *sakazakii* G4023, and plenty of work needs to be done in the future to gain further comprehension.

## Materials and Methods

### Bacterial Strains, Identification, and Growth Conditions

All the strains, plasmids, and primers used in this study are listed in Supplementary Table 6. The *C*. *sakazakii* G4023 strain is a food-isolated strain kindly offered by Beijing Entry--Exit Inspection and Quarantine Bureau and belongs to ST4. The ST type of G4023 was defined by the *Cronobacter* spp. multilocus sequence typing (MLST) Database.^1^ Genomic comparisons between G4023 and ATCC 29544 (ST8) were performed to identify G4023 as a *C*. *sakazakii* strain. The whole genome comparison was performed by local BLASTp (v2.3.0)^2^ with an E-value cutoff of <1e−10, identity >80%, and coverage >80%, and genomic islands were identified by software IslandViewer 4. Then the results were visualized by software BLAST Ring Image Generator (BRIG). The ANI and TUD were calculated *via* online server JspeciesWS^3^ (Richter et al., 2016); The AAI was calculated by software toolkit CompareM.^4^ Overnight LB-cultured bacteria of *C*. *sakazakii* G4023, *C*. *sakazakii* ATCC 29544, EHEC O157: H7 EDL933, and *Salmonella* Typhimurium ATCC 14028 were steak cultured on the selective DFI agar and the LB agar; the plates were then statically cultured in 37°C overnight to observe the phenotypes of colonies. Meanwhile, biochemical identifications of G4023 and ATCC 29544 were performed with commercial biochemical identification kit [Biochemical identification strip of *E. sakazakii* (GB), Hopebio, Qingdao, China], and the positive or negative responses were judged by the color changes of mediums according to the manufacturer’s instructions.

Bacteria in this study were grown at 37°C in LB medium under standard bacterial growth conditions unless otherwise specified. For growth curves, three replicates per strain were grown in 200 μl LB medium in a 96-well plate and inoculated aerobically in the ratio of 1:1,000 from overnight cultures at 37°C. The initial inoculum was approximately 2 × 10^5^ CFU and monitored by measuring the OD_600_ value using a Multiskan GO 1510 plate reader (Thermo Fisher Scientific, Vantaa, FIN) every 10 min.

### Mutant Strain Constructions of *Cronobacter sakazakii*

Mutant strains were generated using the λ Red recombination system described previously (Datta et al., 2006) with the plasmid pSIM17 generated by Chan et al. (2007). The plasmid pSIM17 is a temperature-sensitive, low-copy plasmid and contains blasticidin resistance. In brief, we first electroporated pSIM17 into *C*. *sakazakii* G4023 bacteria and selected on blasticidin-containing (50 μg/ml) LB agars at 30°C. Then the kanamycin cassette used to replace the target genes of *C*. *sakazakii* G4023 was amplified from pKD4 with primers listed in Supplementary Table 6. Each primer contains a 5′ end homologous to the flanking regions of the target gene and a 3′ end that primes the kanamycin cassette for PCR amplification. The amplified PCR products were purified with SanPrep Column DNA Gel Extraction Kit (Sangon Biotech, China) and electroporated into pSIM17-containing *C*. *sakazakii* G4023 strain, which had been induced for red expression by growth at 42°C for 15 min. After electroporation, 1 ml LB was added, and the bacteria were incubated at 37°C with shaking for 3–4 h. Then the bacteria were cultured on LB agar containing 50 μg/ml of kanamycin to select drug resistant recombinants at 37°C. Several colonies on each plate were picked on the following day and amplified with mutant identification primers, after which the amplified products were sequenced to verify the replacement of target genes with kanamycin cassette (Njoroge et al., 2012). The verified mutant colonies were re-cultured in LB medium supplemented with kanamycin (50 μg/ml) at 37°C and preserved in glycerin at −80°C until use.

### Desiccation Tolerance Test

Strains used for desiccation tolerance test were grown overnight and transferred to fresh LB medium at a ratio of 1:100 for 4 h until mid-exponential phase, and 50-μl aliquots were dispensed into individual wells of a 12-well microtiter plate, and three replicates were set for each strain. Thereafter, the bacteria were desiccated as previously described (Shaker et al., 2008; Hu et al., 2017). In brief, the plates were incubated statically in a 37°C incubator without a lid for 4 h for drying. Dehydrated silica gel was placed in the incubator. After drying, the plate was covered with a lid and kept at 30°C statically for 4 days. After desiccation, the cells were rehydrated with 1 ml sterile phosphate buffered saline (PBS, pH 7.4), and 100-μl aliquots were re-cultured in LB agar plates at 37°C for 24 h to count the cell population (CFU/ml).

### RNA Extraction and Sequencing

RNA samples from desiccated *C*. *sakazakii* G4023 were prepared using TRIzol^®^ LS Reagent (Invitrogen, Carlsbad, CA, United States) and treated with RNase-Free DNase I (Fermentas, Burlington, Canada) to eliminate genomic DNA contamination. *C*. *sakazakii* G4023 cultured in LB medium was set as control. Purified RNA samples were stored at -80°C till further use. The library preparation and RNA-seq were carried out commercially (Novogene, Tianjin, China). In brief, a total of 3 μg RNA per sample was used as input material for the RNA sample preparations. Sequencing libraries were generated using NEBNext^®^ Ultra™ Directional RNA Library Prep Kit for Illumina^®^ (NEB, United States) following manufacturer’s recommendations. In order to select cDNA fragments of preferentially 150–200 bp in length, the library fragments were purified with AMPure XP system (Beckman Coulter, Beverly, United States). Then 3 μl USER Enzyme (NEB, United States) was used with size-selected, adaptor-ligated cDNA at 37°C for 15 min followed by 5 min at 95°C before PCR. Then PCR was performed with Phusion High-Fidelity DNA polymerase, Universal PCR primers and Index (X) Primer. At last, products were purified (AMPure XP system), and library quality was assessed on the Agilent Bioanalyzer 2100 system. The clustering of the index-coded samples was performed on a cBot Cluster Generation System using TruSeq PE Cluster Kit v3-cBot-HS (Illumia) according to the manufacturer’s instructions. After cluster generation, the library preparations were sequenced on the NovaSeq 6000 (Illumina), and paired-end reads were generated.

### Protein Extraction and Analysis

For proteomic analysis, desiccated *C*. *sakazakii* G4023 cells were harvested and washed with ice-cold PBS five times. Cells were stored in liquid nitrogen overnight and then stored at −80°C until required. Samples were sonicated three times on ice using a high-intensity ultrasonic processor (Scientz) in lysis buffer (8 M urea, 1% Protease Inhibitor Cocktail). The remaining debris was removed by centrifugation at 12,000 *g* at 4°C for 10 min. Finally, the supernatant was collected, and the protein concentration was determined with BCA Protein Assay Kit (abcam, Cambridge, England) according to the manufacturer’s instructions. For digestion, the protein solution was treated as previously described (Ondrej et al., 2020). Accordingly, the protein solution was reduced with 5 mM dithiothreitol for 30 min at 56°C and alkylated with 11 mM iodoacetamide for 15 min at room temperature in darkness. The protein sample was then diluted by adding 100 mM triethylammonium bicarbonate (TEAB) to urea concentration less than 2 M. Finally, trypsin was added at 1:50 trypsin-to-protein mass ratio for the first digestion overnight and 1:100 trypsin-to-protein mass ratio for a second 4-h digestion. *C*. *sakazakii* G4023 cultured in LB medium was set as control. Three biological replicates were prepared. The tryptic peptides were then submitted to conduct the label-free quantitative mass spectrometry analyses commercially (PTM BIO, Hangzhou, China); high-performance liquid chromatography (HPLC) fractionation and liquid chromatographic-tandem mass spectrometry (LC-MS/MS) analyses were performed as previously described (Ondrej et al., 2020). Briefly, the tryptic peptides were fractionated into fractions by high pH reverse-phase HPLC using Thermo Betasil C18 column [5 μm particles, 10 mm inner diameter (ID), 250 mm length]. Peptides were first separated with a gradient of 8–32% acetonitrile (pH 9.0) over 60 min into 60 fractions. Then, the peptides were combined into six fractions and dried by vacuum centrifuging. The tryptic peptides were then dissolved in 0.1% formic acid (solvent A), directly loaded onto a home-made reversed-phase analytical column (15-cm length, 75-μm ID). The gradient comprised an increase from 6 to 23% solvent B (0.1% formic acid in 98% acetonitrile) over 26 min, 23–35% in 8 min, climbing to 80% in 3 min, and then holding at 80% for the last 3 min, all at a constant flow rate of 400 nl/min on an EASY-nLC 1000 ultra-performance liquid chromatography (UPLC) system. The peptides were subjected to nanospray ionization (NSI) source followed by MS/MS in Q ExactiveTM Plus (Thermo Fisher Scientific) coupled online to the UPLC. The electrospray voltage applied was 2.0 kV. The m/z scan range was 350–1,800 for full scan, and intact peptides were detected in the Orbitrap at a resolution of 70,000. Peptides were then selected for MS/MS using normalized collision energies (NCE) setting of 28, and the fragments were detected in the Orbitrap at a resolution of 17,500. A data-dependent procedure alternated between one MS scan and 20 MS/MS scans with 15.0 s dynamic exclusion. Automatic gain control (AGC) was set at 5E4. Fixed first mass was set at 100 m/z. Finally, the resulting MS/MS spectrums were searched against UniProt database *Cronobacter*_*sakazakii*_strain_ATCC_BAA-894_290339_PR_20191018.

### Quantitative Real-Time PCR Analysis

RNA-seq data were validated using qRT-PCR. RNA purified from the desiccated and control *C*. *sakazakii* G4023 cells was transcribed to cDNA using the PrimeScript 1st Strand cDNA Synthesis Kit (Takara, Japan). qRT-PCR analysis was performed in an Applied Biosystems ABI 7300 Real-Time PCR System. Each qRT-PCR reaction was carried out in a total volume of 20 μl in a 96-well optical reaction plate (Applied Biosystems, Foster, CA, United States) containing 10 μl Power SYBR Green PCR Master Mix (Applied Biosystems, Foster, CA, United States), 1 μl cDNA, and two gene-specific primers with a final concentration of 0.3 mM each. The housekeeping gene *glnS* was used as a reference control for normalization as its transcriptional expression levels did not change dramatically when *C*. *sakazakii* G4023 bacteria suffered desiccation (Supplementary Figure 2). Relative difference in gene expression was calculated as the fold change using the 2^–ΔΔCt^ method. At least three biological replicates were performed for each qRT-PCR analysis.

### Quantitative Biofilm Assay

Crystal violet staining assay was performed to assess the formation of biofilm as described previously (Fernández-Gómez et al., 2020). In brief, 100-μl overnight strains were cultured in 96-well polystyrene microtiter plates and subjected to desiccation. On the third day of desiccation, the 96-well plates were taken out, and overnight-cultured strains were diluted 1:100 in fresh LB medium in the remaining blank wells. The plate was then statically cultured at 30°C for 24 h. The desiccated and LB cultured bacteria were stained with 0.5% crystal violet for 5 min after washing with double-distilled water. Next, 200 μl of 95% ethanol was added after removing the unbound crystal violet to dissolve the biofilm-bound dye, and the ability of biofilm formation was determined by measuring the absorbance at 590 nm with an enzyme-linked immunosorbent assay plate reader. Three wells of each strain were studied in parallel, and each experiment was independently performed thrice.

### Statistical Analysis

Desiccation tolerance test data are presented as the mean ± standard deviation (SD) of at least three trials, and the differences between two groups were evaluated in the independent samples by two-tailed Student’s *t*-test. A *p*-value < 0.05 was considered statistically significant. The asterisks indicate significant differences (*p < 0.05; ^**^p < 0.01; ^***^p < 0.001).

## Data Availability Statement

The raw data of transcriptomes of LB cultured and desiccated *C. sakazakii* G4023 for this study can be found in the GenBank database. The accession number of this BioProject is PRJNA692549 (https://www.ncbi.nlm.nih.gov/bioproject/PRJNA692549).

## Author Contributions

CQ wrote the manuscript. CQ and MH designed and performed the experiments and were involved in the data analysis. YD, JS, and ZY contributed to the bioinformatic analysis. HM helped with the sample preparation for transcriptome and proteome. YW and CY validated the bioinformatic data. SZ helped with the validation of this manuscript. BL (10th author) and BL (11th author) conceptualized and supervised this study. All authors contributed to the article and approved the submitted version.

## Conflict of Interest

The authors declare that the research was conducted in the absence of any commercial or financial relationships that could be construed as a potential conflict of interest.

## Publisher’s Note

All claims expressed in this article are solely those of the authors and do not necessarily represent those of their affiliated organizations, or those of the publisher, the editors and the reviewers. Any product that may be evaluated in this article, or claim that may be made by its manufacturer, is not guaranteed or endorsed by the publisher.

## Footnotes

1
https://pubmlst.org/organisms/cronobacter-spp

2
https://ftp.ncbi.nlm.nih.gov/blast/executables/blast+/LATEST/

3
http://jspecies.ribohost.com/jspeciesws/

4
https://github.com/dparks1134/CompareM

## References

[B2] Almajed F. S. Forsythe S. J. (2016). *Cronobacter sakazakii* clinical isolates overcome host barriers and evade the immune response. *Microb. Pathog.* 90 55–63. 10.1016/j.micpath.2015.11.014 26616163

[B3] Ayala-Castro C. Saini A. Outten F. W. (2008). Fe-S cluster assembly pathways in bacteria. *Microbiol. Mol. Biol. Rev.* 72 110–125. 10.1128/MMBR.00034-07 18322036 PMC2268281

[B4] Bai H. Zhou D. Zhang X. Cao Y. Xiao X. Zhang Y. (2021). The responses of *Salmonella enterica* serovar Typhimurium to vanillin in apple juice through global transcriptomics. *Int. J. Food Microbiol.* 347:109189. 10.1016/j.ijfoodmicro.2021.109189 33838479

[B5] Bayles D. O. Wilkinson B. J. (2000). Osmoprotectants and cryoprotectants for *Listeria monocytogenes*. *Lett. Appl. Microbiol.* 30 23–27. 10.1046/j.1472-765x.2000.00646.x 10728555

[B6] Bennour Hennekinne R. Guillier L. Fazeuilh L. Ells T. Forsythe S. Jackson E. (2018). Survival of Cronobacter in powdered infant formula and their variation in biofilm formation. *Lett. Appl. Microbiol.* 66 496–505. 10.1111/lam.12879 29575083

[B7] Berleman J. E. Bauer C. E. (2005). A che-like signal transduction cascade involved in controlling flagella biosynthesis in *Rhodospirillum centenum*. *Mol. Microbiol.* 55 1390–1402. 10.1111/j.1365-2958.2005.04489.x 15720548

[B8] Buccitelli C. Selbach M. (2020). mRNAs, proteins and the emerging principles of gene expression control. *Nat. Rev. Genet.* 21 630–644. 10.1038/s41576-020-0258-4 32709985

[B9] Caldara M. Dupont G. Leroy F. Goldbeter A. De Vuyst L. Cunin R. (2008). Arginine biosynthesis in *Escherichia coli*: experimental perturbation and mathematical modeling. *J. Biol. Chem.* 283 6347–6358. 10.1074/jbc.M705884200 18165237

[B10] Cawthorn D. M. Botha S. Witthuhn R. C. (2008). Evaluation of different methods for the detection and identification of *Enterobacter sakazakii* isolated from South African infant formula milks and the processing environment. *Int. J. Food Microbiol.* 127 129–138. 10.1016/j.ijfoodmicro.2008.06.024 18687498

[B11] Chaibenjawong P. Foster S. J. (2011). Desiccation tolerance in *Staphylococcus aureus*. *Arch. Microbiol.* 193 125–135. 10.1007/s00203-010-0653-x 21088825

[B12] Chan W. Costantino N. Li R. Lee S. C. Su Q. Melvin D. (2007). A recombineering based approach for high-throughput conditional knockout targeting vector construction. *Nucleic Acids Res.* 35:e64. 10.1093/nar/gkm163 17426124 PMC1885671

[B13] Chauhan R. Singh N. Pal G. K. Goel G. (2020). Trending biocontrol strategies against *Cronobacter sakazakii*: a recent updated review. *Food Res. Int.* 137:109385. 10.1016/j.foodres.2020.109385 33233087

[B14] Chen G. Gharib T. G. Huang C. C. Taylor J. M. Misek D. E. Kardia S. L. (2002). Discordant protein and mRNA expression in lung adenocarcinomas. *Mol. Cell. Proteomics* 1 304–313. 10.1074/mcp.m200008-mcp200 12096112

[B15] da Silva P. Manieri F. Z. Herrera C. M. Trent M. S. Moreira C. G. (2018). Novel role of VisP and the Wzz system during O-antigen assembly in *Salmonella enterica* serovar Typhimurium pathogenesis. *Infect. Immun.* 86:e00319-18. 10.1128/iai.00319-18 29866904 PMC6056878

[B16] Datta S. Costantino N. Court D. L. (2006). A set of recombineering plasmids for gram-negative bacteria. *Gene* 379 109–115. 10.1016/j.gene.2006.04.018 16750601

[B17] Du X. J. Wang X. Y. Dong X. Li P. Wang S. (2018). Characterization of the desiccation tolerance of *Cronobacter sakazakii* strains. *Front. Microbiol.* 9:2867. 10.3389/fmicb.2018.02867 30542333 PMC6278591

[B18] Farmer J. J. Asbury M. Hickman F. Brenner D. J. Enterobacteriaceae Study Group (1980). *Enterobacter sakazakii*: a new species of “Enterobacteriaceae” isolated from clinical specimens. *Int. J. Syst. Evol. Microbiol.* 30 569–584. 10.1099/00207713-30-3-569

[B19] Fernández-Gómez P. López M. Prieto M. González-Raurich M. Alvarez-Ordóñez A. (2020). The role of the general stress response regulator RpoS in *Cronobacter sakazakii* biofilm formation. *Food Res. Int.* 136:109508. 10.1016/j.foodres.2020.109508 32846586

[B20] Garmiri P. Coles K. E. Humphrey T. J. Cogan T. A. (2008). Role of outer membrane lipopolysaccharides in the protection of *Salmonella enterica* serovar Typhimurium from desiccation damage. *FEMS Microbiol. Lett.* 281 155–159. 10.1111/j.1574-6968.2008.01093.x 18312578

[B21] Ghazalpour A. Bennett B. Petyuk V. A. Orozco L. Hagopian R. Mungrue I. N. (2011). Comparative analysis of proteome and transcriptome variation in mouse. *PLoS Genet.* 7:e1001393. 10.1371/journal.pgen.1001393 21695224 PMC3111477

[B22] Gibson D. L. White A. P. Snyder S. D. Martin S. Heiss C. Azadi P. (2006). *Salmonella* produces an O-antigen capsule regulated by AgfD and important for environmental persistence. *J. Bacteriol.* 188 7722–7730. 10.1128/JB.00809-06 17079680 PMC1636306

[B23] Gronow S. Brade H. (2001). Lipopolysaccharide biosynthesis: which steps do bacteria need to survive? *J. Endotoxin Res.* 7 3–23. 11521077

[B24] Haider S. Pal R. (2013). Integrated analysis of transcriptomic and proteomic data. *Curr. Genom.* 14 91–110. 10.2174/1389202911314020003 PMC3637682 24082820

[B25] Han C. L. Chien C. W. Chen W. C. Chen Y. R. Wu C. P. Li H. (2008). A multiplexed quantitative strategy for membrane proteomics: opportunities for mining therapeutic targets for autosomal dominant polycystic kidney disease. *Mol. Cell Proteomics* 7 1983–1997. 10.1074/mcp.M800068-MCP200 18490355

[B26] Hansen L. T. Vogel B. F. (2011). Desiccation of adhering and biofilm *Listeria monocytogenes* on stainless steel: survival and transfer to salmon products. *Int. J. Food Microbiol.* 146 88–93. 10.1016/j.ijfoodmicro.2011.01.032 21334756

[B27] He K. Bauer C. E. (2014). Chemosensory signaling systems that control bacterial survival. *Trends Microbiol.* 22 389–398. 10.1016/j.tim.2014.04.004 24794732 PMC4273944

[B28] Helbig A. O. Heck A. J. Slijper M. (2010). Exploring the membrane proteome–challenges and analytical strategies. *J. Proteomics* 73 868–878. 10.1016/j.jprot.2010.01.005 20096812

[B29] Hu S. Yu Y. Wu X. Xia X. Xiao X. Wu H. (2017). Comparative proteomic analysis of *Cronobacter sakazakii* by iTRAQ provides insights into response to desiccation. *Food Res Int* 100(Pt 1) 631–639. 10.1016/j.foodres.2017.06.051 28873731

[B30] Hughes K. T. Mathee K. (1998). The anti-sigma factors. *Annu. Rev. Microbiol.* 52 231–286. 10.1146/annurev.micro.52.1.231 9891799

[B31] Iversen C. Lehner A. Mullane N. Bidlas E. Cleenwerck I. Marugg J. (2007). The taxonomy of *Enterobacter sakazakii*: proposal of a new genus *Cronobacter* gen. nov. and descriptions of *Cronobacter sakazakii* comb. nov. *Cronobacter sakazakii* subsp. *sakazakii*, comb. nov., *Cronobacter sakazakii* subsp. *malonaticus* subsp. nov., *Cronobacter turicensis* sp. nov., *Cronobacter muytjensii* sp. nov., *Cronobacter dublinensis* sp. nov. and *Cronobacter genomospecies* 1. *BMC Evol. Biol.* 7:64. 10.1186/1471-2148-7-64 17439656 PMC1868726

[B32] Iversen C. Mullane N. McCardell B. Tall B. D. Lehner A. Fanning S. (2008). Cronobacter gen. nov., a new genus to accommodate the biogroups of *Enterobacter sakazakii*, and proposal of *Cronobacter sakazakii* gen. nov., comb. nov., *Cronobacter malonaticus* sp. nov., *Cronobacter turicensis* sp. nov., *Cronobacter muytjensii* sp. nov., *Cronobacter dublinensis* sp. nov., *Cronobacter genomospecies* 1, and of three subspecies, *Cronobacter dublinensis* subsp. dublinensis subsp. nov., *Cronobacter dublinensis* subsp. *lausannensis* subsp. nov. and *Cronobacter dublinensis* subsp. *lactaridi* subsp. nov. *Int. J. Syst. Evol. Microbiol.* 58(Pt 6) 1442–1447. 10.1099/ijs.0.65577-0 18523192

[B33] Joseph S. Cetinkaya E. Drahovska H. Levican A. Figueras M. J. Forsythe S. J. (2012). *Cronobacter condimenti* sp. nov., isolated from spiced meat, and *Cronobacter universal*is sp. nov., a species designation for *Cronobacter* sp. genomospecies 1, recovered from a leg infection, water and food ingredients. *Int. J. Syst. Evol. Microbiol.* 62(Pt 6) 1277–1283. 10.1099/ijs.0.032292-0 22661070

[B34] Kapatral V. Olson J. W. Pepe J. C. Miller V. L. Minnich S. A. (1996). Temperature-dependent regulation of *Yersinia enterocolitica* class III flagellar genes. *Mol. Microbiol.* 19 1061–1071. 10.1046/j.1365-2958.1996.452978.x 8830263

[B35] Kehry M. R. Doak T. G. Dahlquist F. W. (1984). Stimulus-induced changes in methylesterase activity during chemotaxis in *Escherichia coli*. *J. Biol. Chem.* 259 11828–11835. 6384215

[B36] Kintz E. N. Goldberg J. B. (2011). Site-directed mutagenesis reveals key residue for O antigen chain length regulation and protein stability in *Pseudomonas aeruginosa* Wzz2. *J. Biol. Chem.* 286 44277–44284. 10.1074/jbc.M111.273979 22069314 PMC3243511

[B37] Kutsukake K. Iino T. (1994). Role of the FliA-FlgM regulatory system on the transcriptional control of the flagellar regulon and flagellar formation in *Salmonella typhimurium*. *J. Bacteriol.* 176 3598–3605. 10.1128/jb.176.12.3598-3605.1994 8206838 PMC205549

[B38] Lai Y. Zhang D. Wang J. Wang J. Ren P. Yao L. (2020). Integrative transcriptomic and proteomic analyses of molecular mechanism responding to salt stress during seed germination in hulless barley. *Int. J. Mol. Sci.* 21:359. 10.3390/ijms21010359 31935789 PMC6981547

[B39] Li H. Bhaskara A. Megalis C. Tortorello M. L. (2012). Transcriptomic analysis of *Salmonella* desiccation resistance. *Foodborne Pathog. Dis.* 9 1143–1151. 10.1089/fpd.2012.1254 23237410

[B40] Liu B. Furevi A. Perepelov A. V. Guo X. Cao H. Wang Q. (2019). Structure and genetics of *Escherichia coli* O antigens. *FEMS Microbiol. Rev.* 44 655–683. 10.1093/femsre/fuz028 31778182 PMC7685785

[B41] MacKay V. L. Li X. Flory M. R. Turcott E. Law G. L. Serikawa K. A. (2004). Gene expression analyzed by high-resolution state array analysis and quantitative proteomics: response of yeast to mating pheromone. *Mol. Cell. Proteomics* 3 478–489. 10.1074/mcp.M300129-MCP200 14766929

[B42] Maier T. Guell M. Serrano L. (2009). Correlation of mRNA and protein in complex biological samples. *FEBS Lett.* 583 3966–3973. 10.1016/j.febslet.2009.10.036 19850042

[B43] Muok A. R. Briegel A. Crane B. R. (2020). Regulation of the chemotaxis histidine kinase CheA: a structural perspective. *Biochim. Biophys. Acta Biomembr.* 1862:183030. 10.1016/j.bbamem.2019.183030 31374212 PMC7212787

[B44] Murray G. L. Attridge S. R. Morona R. (2003). Regulation of *Salmonella typhimurium* lipopolysaccharide O antigen chain length is required for virulence; identification of FepE as a second Wzz. *Mol. Microbiol.* 47 1395–1406. 10.1046/j.1365-2958.2003.03383.x 12603743

[B45] Njoroge J. W. Nguyen Y. Curtis M. M. Moreira C. G. Sperandio V. (2012). Virulence meets metabolism: Cra and KdpE gene regulation in enterohemorrhagic *Escherichia coli*. *mBio* 3:e00280-12. 10.1128/mBio.00280-12 23073764 PMC3482499

[B46] Okuda S. Sherman D. J. Silhavy T. J. Ruiz N. Kahne D. (2016). Lipopolysaccharide transport and assembly at the outer membrane: the PEZ model. *Nat. Rev. Microbiol.* 14 337–345. 10.1038/nrmicro.2016.25 27026255 PMC4937791

[B47] Ondrej M. Rehulka P. Rehulkova H. Kupcik R. Tichy A. (2020). Fractionation of enriched phosphopeptides using pH/acetonitrile-gradient-reversed-phase microcolumn separation in combination with LC-MS/MS analysis. *Int. J. Mol. Sci.* 21:3971. 10.3390/ijms21113971 32492839 PMC7312998

[B48] Osaili T. Forsythe S. (2009). Desiccation resistance and persistence of *Cronobacter* species in infant formula. *Int. J. Food Microbiol.* 136 214–220. 10.1016/j.ijfoodmicro.2009.08.006 19720413

[B49] Ozenberger B. A. Nahlik M. S. McIntosh M. A. (1987). Genetic organization of multiple fep genes encoding ferric enterobactin transport functions in *Escherichia coli*. *J. Bacteriol.* 169 3638–3646. 10.1128/jb.169.8.3638-3646.1987 2956250 PMC212444

[B50] Parra-Flores J. Aguirre J. Juneja V. Jackson E. E. Cruz-Córdova A. Silva-Sanchez J. (2018). Virulence and antibiotic resistance profiles of *Cronobacter sakazakii* and *Enterobacter* spp. Involved in the diarrheic hemorrhagic outbreak in Mexico. *Front. Microbiol.* 9:2206. 10.3389/fmicb.2018.02206 30319560 PMC6171480

[B51] Parra-Flores J. Maury-Sintjago E. Rodriguez-Fernández A. Acuña S. Cerda F. Aguirre J. (2020). Microbiological quality of powdered infant formula in Latin America. *J. Food Prot.* 83 534–541. 10.4315/0362-028x.jfp-19-399 32078682

[B52] Richter M. Rosselló-Móra R. Oliver Glöckner F. Peplies J. (2016). JSpeciesWS: a web server for prokaryotic species circumscription based on pairwise genome comparison. *Bioinformatics* 32 929–931. 10.1093/bioinformatics/btv681 26576653 PMC5939971

[B53] Roche B. Aussel L. Ezraty B. Mandin P. Py B. Barras F. (2013). Iron/sulfur proteins biogenesis in prokaryotes: formation, regulation and diversity. *Biochim. Biophys. Acta* 1827 455–469. 10.1016/j.bbabio.2012.12.010 23298813

[B54] Romilly C. Hoekzema M. Holmqvist E. Wagner E. G. H. (2020). Small RNAs OmrA and OmrB promote class III flagellar gene expression by inhibiting the synthesis of anti-Sigma factor FlgM. *RNA Biol.* 17 872–880. 10.1080/15476286.2020.1733801 32133913 PMC7549644

[B55] Sandberg A. Branca R. M. Lehtio J. Forshed J. (2014). Quantitative accuracy in mass spectrometry based proteomics of complex samples: the impact of labeling and precursor interference. *J. Proteomics* 96 133–144. 10.1016/j.jprot.2013.10.035 24211767

[B56] Schmidt H. Hensel M. (2004). Pathogenicity islands in bacterial pathogenesis. *Clin. Microbiol. Rev.* 17 14–56. 10.1128/cmr.17.1.14-56.2004 14726454 PMC321463

[B57] Shaker R. R. Osaili T. M. Abu Al-Hasan A. S. Ayyash M. M. Forsythe S. J. (2008). Effect of desiccation, starvation, heat, and cold stresses on the thermal resistance of *Enterobacter sakazakii* in rehydrated infant milk formula. *J. Food Sci.* 73 M354–M359. 10.1111/j.1750-3841.2008.00880.x 18803719

[B58] Srikumar S. Cao Y. Yan Q. Van Hoorde K. Nguyen S. Cooney S. (2019). RNA sequencing-based transcriptional overview of xerotolerance in *Cronobacter sakazakii* SP291. *Appl. Environ. Microbiol.* 85:e01993-18. 10.1128/aem.01993-18 30446557 PMC6344630

[B59] Stephens B. B. Loar S. N. Alexandre G. (2006). Role of CheB and CheR in the complex chemotactic and aerotactic pathway of *Azospirillum brasilens*e. *J. Bacteriol.* 188 4759–4768. 10.1128/JB.00267-06 16788185 PMC1483015

[B60] Sun Y. Wang M. Liu H. Wang J. He X. Zeng J. (2011). Development of an O-antigen serotyping scheme for *Cronobacter sakazakii*. *Appl. Environ. Microbiol.* 77 2209–2214. 10.1128/aem.02229-10 21296934 PMC3067421

[B61] Toker A. S. Macnab R. M. (1997). Distinct regions of bacterial flagellar switch protein FliM interact with FliG, FliN and CheY. *J. Mol. Biol.* 273 623–634. 10.1006/jmbi.1997.1335 9356251

[B62] Trinh H. V. Grossmann J. Gehrig P. Roschitzki B. Schlapbach R. Greber U. F. (2013). iTRAQ-based and label-free proteomics approaches for studies of human adenovirus infections. *Int. J. Proteomics* 2013:581862. 10.1155/2013/581862 23555056 PMC3608280

[B63] Vanderlinde E. M. Harrison J. J. Muszynski A. Carlson R. W. Turner R. J. Yost C. K. (2010). Identification of a novel ABC transporter required for desiccation tolerance, and biofilm formation in *Rhizobium leguminosarum* bv. viciae 3841. *FEMS Microbiol. Ecol.* 71 327–340. 10.1111/j.1574-6941.2009.00824.x 20030718 PMC2868943

[B64] Vyas S. Maas W. K. (1963). Feedback inhibition of acetylglutamate synthetase by arginine in *Escherichia coli*. *Arch. Biochem. Biophys.* 100 542–546. 10.1016/0003-9861(63)90124-3 13998082

[B65] Wang H. Alvarez S. Hicks L. M. (2012). Comprehensive comparison of iTRAQ and label-free LC-based quantitative proteomics approaches using two *Chlamydomonas reinhardtii* strains of interest for biofuels engineering. *J. Proteome Res.* 11 487–501. 10.1021/pr2008225 22059437

[B66] Wang L. Hu X. Tao G. Wang X. (2012). Outer membrane defect and stronger biofilm formation caused by inactivation of a gene encoding for heptosyltransferase I in *Cronobacter sakazakii* ATCC BAA-894. *J. Appl. Microbiol.* 112 985–997. 10.1111/j.1365-2672.2012.05263.x 22353600

[B67] Wang M. Cao B. Gao Q. Sun Y. Liu P. Feng L. (2009). Detection of *Enterobacter sakazakii* and other pathogens associated with infant formula powder by use of a DNA microarray. *J. Clin. Microbiol.* 47 3178–3184. 10.1128/jcm.00366-09 19641057 PMC2756907

[B68] Wang X. Liang H. Guo D. Guo L. Duan X. Jia Q. (2019). Integrated analysis of transcriptomic and proteomic data from tree peony (*P. ostii*) seeds reveals key developmental stages and candidate genes related to oil biosynthesis and fatty acid metabolism. *Hortic. Res.* 6:111. 10.1038/s41438-019-0194-7 31645965 PMC6804530

[B69] Wang M. Wang L. Wu P. Chen T. Zhu Y. Zhang Y. (2019). Genomics and experimental analysis reveal a novel factor contributing to the virulence of *Cronobacter sakazakii* strains associated with neonate infection. *J. Infect. Dis.* 220 306–315. 10.1093/infdis/jiz098 30835279

[B70] White A. P. Gibson D. L. Kim W. Kay W. W. Surette M. G. (2006). Thin aggregative fimbriae and cellulose enhance long-term survival and persistence of *Salmonella*. *J. Bacteriol.* 188 3219–3227. 10.1128/JB.188.9.3219-3227.2006 16621814 PMC1447457

[B71] Wuichet K. Zhulin I. B. (2010). Origins and diversification of a complex signal transduction system in prokaryotes. *Sci. Signal.* 3:ra50. 10.1126/scisignal.2000724 20587806 PMC3401578

[B72] Yan Q. Q. Condell O. Power K. Butler F. Tall B. D. Fanning S. (2012). *Cronobacter* species (formerly known as *Enterobacter sakazakii*) in powdered infant formula: a review of our current understanding of the biology of this bacterium. *J. Appl. Microbiol.* 113 1–15. 10.1111/j.1365-2672.2012.05281.x 22420458

[B73] Yang J. Bitoun J. P. Ding H. (2006). Interplay of IscA and IscU in biogenesis of iron-sulfur clusters. *J. Biol. Chem.* 281 27956–27963. 10.1074/jbc.M601356200 16877383

[B74] Yang J. He Y. Jiang J. Chen W. Gao Q. Pan L. (2016). Comparative proteomic analysis by iTRAQ-2DLC-MS/MS provides insight into the key proteins involved in *Cronobacter* sp. biofilm formation. *Food Control* 63 93–100. 10.1016/j.foodcont.2015.11.029

