# Supplementary material for: Chemotaxis and Shorter O-Antigen Chain Length Contribute to the Strong Desiccation Tolerance of a Food-Isolated Cronobacter sakazakii Strain
Source: Front Microbiol. 2022 Jan 4;12:779538. doi: 10.3389/fmicb.2021.779538 (PMC8764414; doi:10.3389/fmicb.2021.779538)
Supplement: Supplementary file 1 [file Table_1.DOCX]

Table S1. Genetic characteristics of the chromosome of *C. sakazakii* G4023 and ATCC 29544.

|  | **G4023** | **ATCC 29544** |
| --- | --- | --- |
| **Size (bp)** | 4,380,595 | 4,511,265 |
| **No. of CDSs** | 4010 | 4419 |
| **Mean of CDS length (bp)** | 950.9 | 907.0 |
| **No. of rRNA genes** | 22 | 22 |
| **No. of tRNA genes** | 82 | 84 |
| **GC content (%)** | 56.7 | 56.8 |
