# Supplementary material for: Chemotaxis and Shorter O-Antigen Chain Length Contribute to the Strong Desiccation Tolerance of a Food-Isolated Cronobacter sakazakii Strain
Source: Front Microbiol. 2022 Jan 4;12:779538. doi: 10.3389/fmicb.2021.779538 (PMC8764414; doi:10.3389/fmicb.2021.779538)
Supplement: Supplementary file 6 [file Table_6.DOCX]

Table S6. Strains, plasmids, and primers used in this study

| **Strains** | | | |
| --- | --- | --- | --- |
| G4023 | food-isolated wild-type *Cronobacter* *sakazakii* | | Lab collection |
| ATCC 29544 | *Cronobacter* *sakazakii* type-strain | | ATCC |
| Δ*cheY* | *cheY* mutant strain of G4023 | | This study |
| Δ*cheB* | *cheB* mutant strain of G4023 | | This study |
| Δ*cheR* | *cheR* mutant strain of G4023 | | This study |
| Δ*cheW* | *cheW* mutant strain of G4023 | | This study |
| Δ*cheA* | *cheA* mutant strain of G4023 | | This study |
| Δ*fepE* | *fepE* mutant strain of G4023 | | This study |
| Δ*wzzB* | *wzzB* mutant strain of G4023 | | This study |
| **Plasmids** | | | |
| pKD4 | Containing a kanamycin resistance cassette and the flipase recognition sites, KmR | | Lab collection |
| pSIM17 | Red recombination plasmid, BlasticidinR | | Lab collection |
| **Primers for gene mutation (5’--3’)** | | | |
| Δ*cheY* | F | ATGGCGGATAAAGATCTCAAGTTTCTGGTTGTGGATGAgtgtaggctggagctgcttcg | |
|  | R | TCACATGCCGAGTTTTTCGAAAATCTTGCTCAGTTTTTCcatatgaatatcctccttag | |
| Δ*cheB* | F | ATGAGTAAAATCAGAGTGTTGTCAGTAGATGATTCGGCgtgtaggctggagctgcttcg | |
|  | R | TTAAATACGTATTGCCTGTCCGGCACTGATTTTCGCCAGcatatgaatatcctccttag | |
| Δ*cheR* | F | ATGACATCATCCATGCCTGCTGGGCAAACGTCATTATTgtgtaggctggagctgcttcg | |
|  | R | TCATTCTTTTTCCTTACTCAGGGCATACACCGTCTGACCcatatgaatatcctccttag | |
| Δ*cheW* | F | ATGACCGGTATGAGCAACGTCACTAAACTGGCCGGCGAgtgtaggctggagctgcttcg | |
|  | R | TCACGCCACGCGGGCGGTTGCGCTGTCAATCAGCTCCATcatatgaatatcctccttag | |
| Δ*cheA* | F | GTGAGTATGGATATTAGCGATTTTTATCAGACATTTTTgtgtaggctggagctgcttcg | |
|  | R | TCAGGCGGCGGTCGGCGCCAGACGTTGCTCACGGTTTAAcatatgaatatcctccttag | |
| Δ*fepE* | F | ATGTCATCGATAGATATCAAAAATAATCCGCTCACTTCgtgtaggctggagctgcttcg | |
|  | R | TCAGGAGAGCAGCGCGTCGACGTCGCTTGCGTGCTGCGTcatatgaatatcctccttag | |
| Δ*wzzB* | F | ATGAGCAAAGAATTTCAGGGTGACGCTTTTCACCGCAGgtgtaggctggagctgcttcg | |
|  | R | TTAGCGCACTCTGTTGGCGCTGTGTTCGCGAATCGCGTTcatatgaatatcctccttag | |
| **Primers for identification of mutant strains** | | | |
| Δ*cheY* | F | TGTTGTTTCACTCCGTAGCC | |
|  | R | GTCGCTTTCCAGTTTGTTCT | |
| Δ*cheB* | F | GCTGGAAAGTCTACGGCAC | |
|  | R | ACCATCTTCCGCTTCTTCTAC | |
| Δ*cheR* | F | CAAACGAACAGAGTCGGATG | |
|  | R | TCAACATCCAGCGTTAAGACA | |
| Δ*cheW* | F | GTCGGTTAAGGTAAACGAAGAA | |
|  | R | CCTCAAACTCAAAACGGAAG | |
| Δ*cheA* | F | CCGCATAAGTTTGCTGGTAC | |
|  | R | AGGTCAACGATAGGCACGA | |
| Δ*fepE* | F | CGGTGATACAACTTTTGGTAGG | |
|  | R | AATGATGGCGGTGGAAGT | |
| Δ*wzzB* | F | CGAGTGGATGGAATAAGGTT | |
|  | R | CTGGTGAACGAAGCCTCTG | |
| **Primers for qRT-PCR** | | | |
| *glnS* | F | GATCGCAGCGTGGAAGAA | |
|  | R | GCGTACAGAGCGAATGGG | |
| *puuR* | F | TCAGCACCATTGAACAGGAC | |
|  | R | GCGGATTCCCGTTATGAA | |
| *ostA* | F | TGCTGGCGGATAAACTCA | |
|  | R | GCTCCAGAAGTTCGGTGTT | |
| *proV* | F | AAGATAGCGATGGTATTCCAGT | |
|  | R | CCATTAACAGGATGTCAGGG | |
| *cpxA* | F | CTGGGGCGAAGTCCTTG | |
|  | R | GCCTCGTCAGTGCGATAGA | |
| *marR* | F | GCCTAACCCTAACGATAAACG | |
|  | R | GCAGCACCCTTTTAAGCAG | |
| *ompA* | F | GCTTTATCCCTAACGACGG | |
|  | R | GAGGAGTCAGCACGCCATA | |
| *wzy* | F | TCTATTGTTTGCGATGGTGAG | |
|  | R | ACTTCCTCCAATCGTTTTCG | |
| *crtE* | F | GAACGCTAACGCCGTGA | |
|  | R | GATCCATCAACGCAGGCT | |
| *walR* | F | TCGAAAGCGATCTCCAGG | |
|  | R | CGGTCGAGCGGGAAA | |
| *mcbR* | F | GCTCACCTGCGTGAACTCT | |
|  | R | ATCGTCGGACTGCGGATA | |
