# Supplementary material for: Chemotaxis and Shorter O-Antigen Chain Length Contribute to the Strong Desiccation Tolerance of a Food-Isolated Cronobacter sakazakii Strain
Source: Front Microbiol. 2022 Jan 4;12:779538. doi: 10.3389/fmicb.2021.779538 (PMC8764414; doi:10.3389/fmicb.2021.779538)
Supplement: Supplementary file 7 [file Data_Sheet_1.PDF]

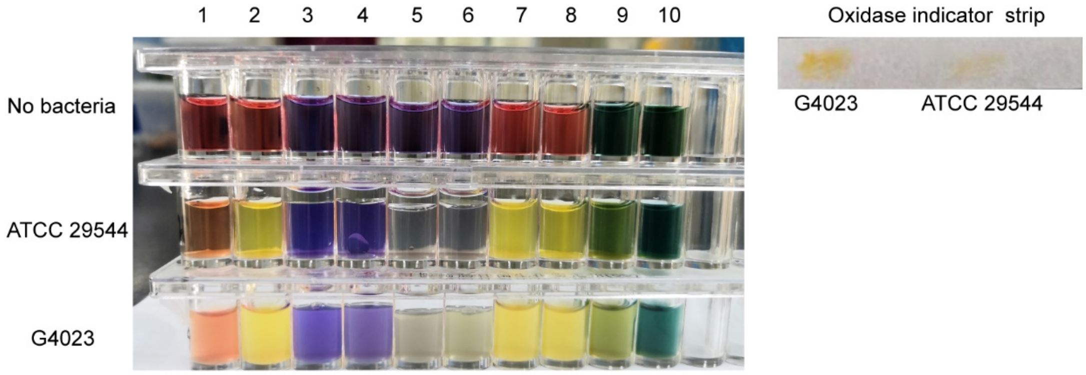

Supplementary Figure 1. Identification of G4023 and ATCC 29544 with commercial identification kit for C. sakazakii species. 1-10 represents mediums supplemented with different ingredients separately. 1: D-Sorbitol; 2: L-rhamnose; 3: L-arginine dihydrolase; 4: ornithine decarboxylase broth; 5: lysine decarboxylase broth; 6: amino acid decarboxylase control; 7: D-sucrose; 8: D-melibiose; 9: amygdalin; 10: Simmons citrate.
