# Supplementary material for: Chemotaxis and Shorter O-Antigen Chain Length Contribute to the Strong Desiccation Tolerance of a Food-Isolated Cronobacter sakazakii Strain
Source: Front Microbiol. 2022 Jan 4;12:779538. doi: 10.3389/fmicb.2021.779538 (PMC8764414; doi:10.3389/fmicb.2021.779538)
Supplement: Supplementary file 8 [file Data_Sheet_2.PDF]

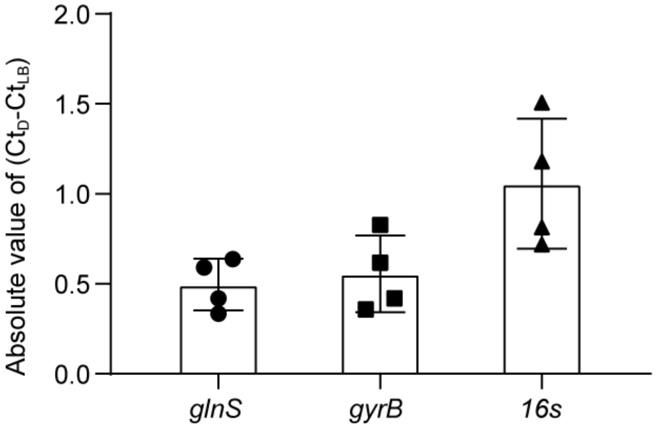

Supplementary Figure 2. Absolute values of Ct values change of *glnS*, *gyrB*, and *16s* in desiccated *C*. *sakazakii* compared with that in LB-cultured.
