# Supplementary material for: Chemotaxis and Shorter O-Antigen Chain Length Contribute to the Strong Desiccation Tolerance of a Food-Isolated Cronobacter sakazakii Strain
Source: Front Microbiol. 2022 Jan 4;12:779538. doi: 10.3389/fmicb.2021.779538 (PMC8764414; doi:10.3389/fmicb.2021.779538)
Supplement: Supplementary file 9 [file Data_Sheet_3.PDF]

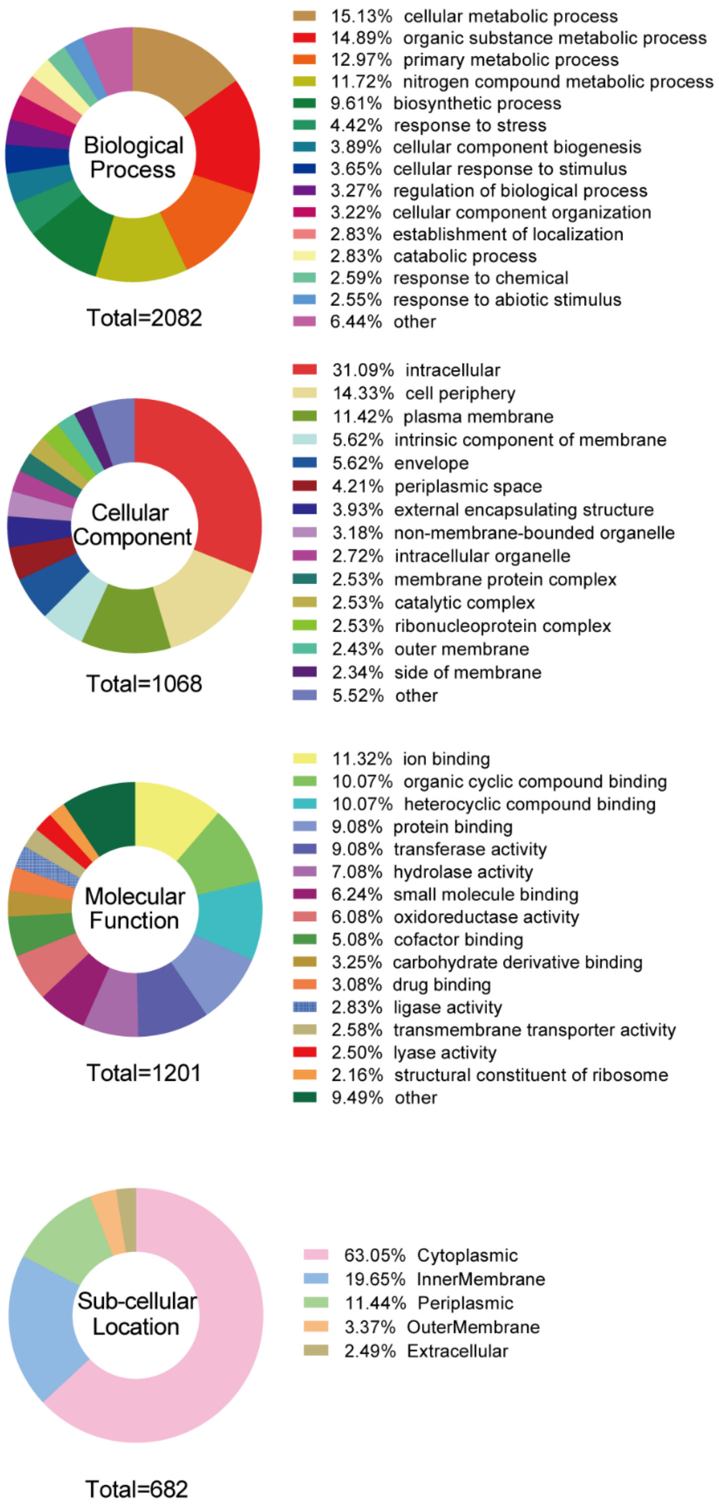

Supplementary Figure 3. Distributions of DEPs in sub-categories of BP, CC, and MF, and sub-cellular location distributions of DEPs.
