# Supplementary material for: Chemotaxis and Shorter O-Antigen Chain Length Contribute to the Strong Desiccation Tolerance of a Food-Isolated Cronobacter sakazakii Strain
Source: Front Microbiol. 2022 Jan 4;12:779538. doi: 10.3389/fmicb.2021.779538 (PMC8764414; doi:10.3389/fmicb.2021.779538)
Supplement: Supplementary file 10 [file Data_Sheet_4.PDF]

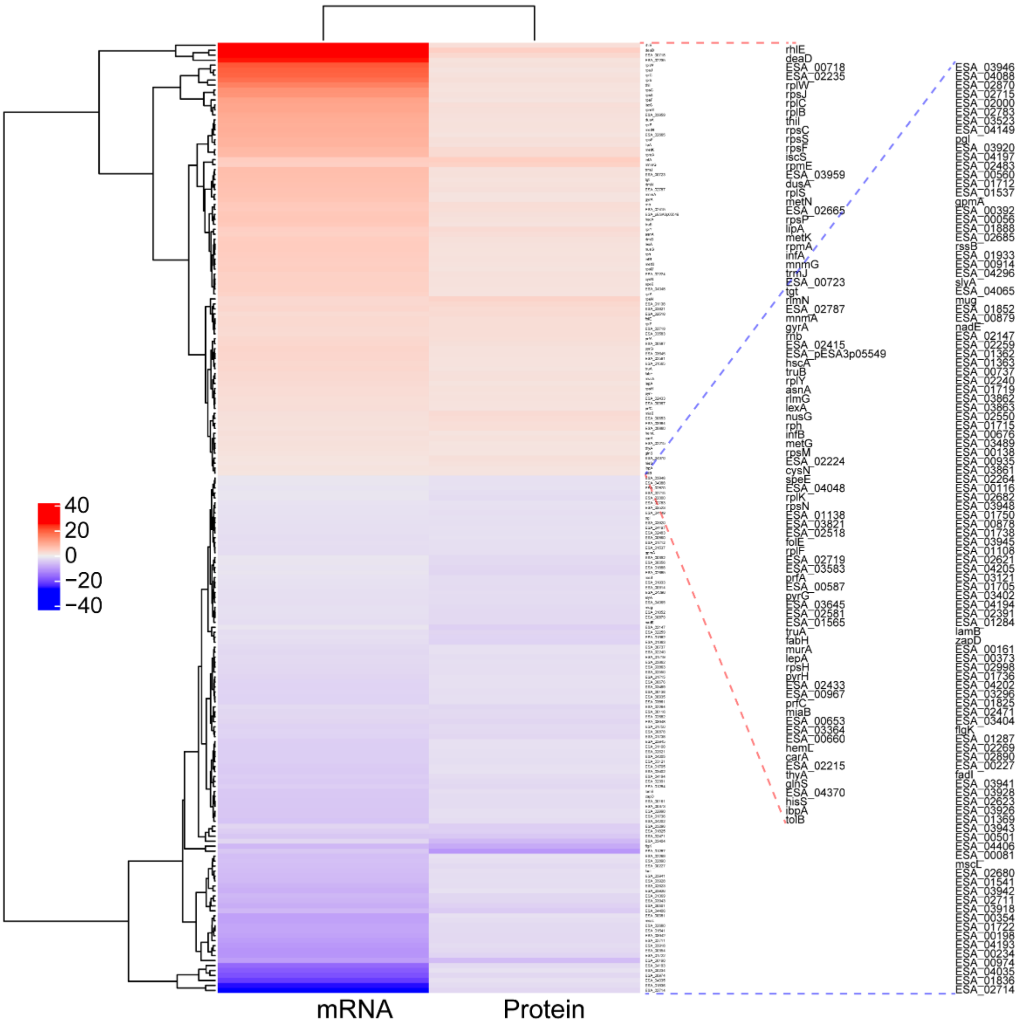

Supplementary Figure 4. Heatmap of expression levels of the 191 genes having consistent regulation trends in transcriptome and proteome. Red represents up-regulated and blue represents down-regulated.
